# Supplementary material for: SLAP controls mTORC2 integrity via UBE3C-mediated non-degradative mLST8 ubiquitination to suppress colorectal tumorigenesis
Source: Cell Death Differ. 2025 Dec 15;33(5):1020–35. doi: 10.1038/s41418-025-01633-1 (PMC13156305; doi:10.1038/s41418-025-01633-1)
Supplement: Supplementary file 1 — Supplementary Figures [file 41418_2025_1633_MOESM1_ESM.pdf]

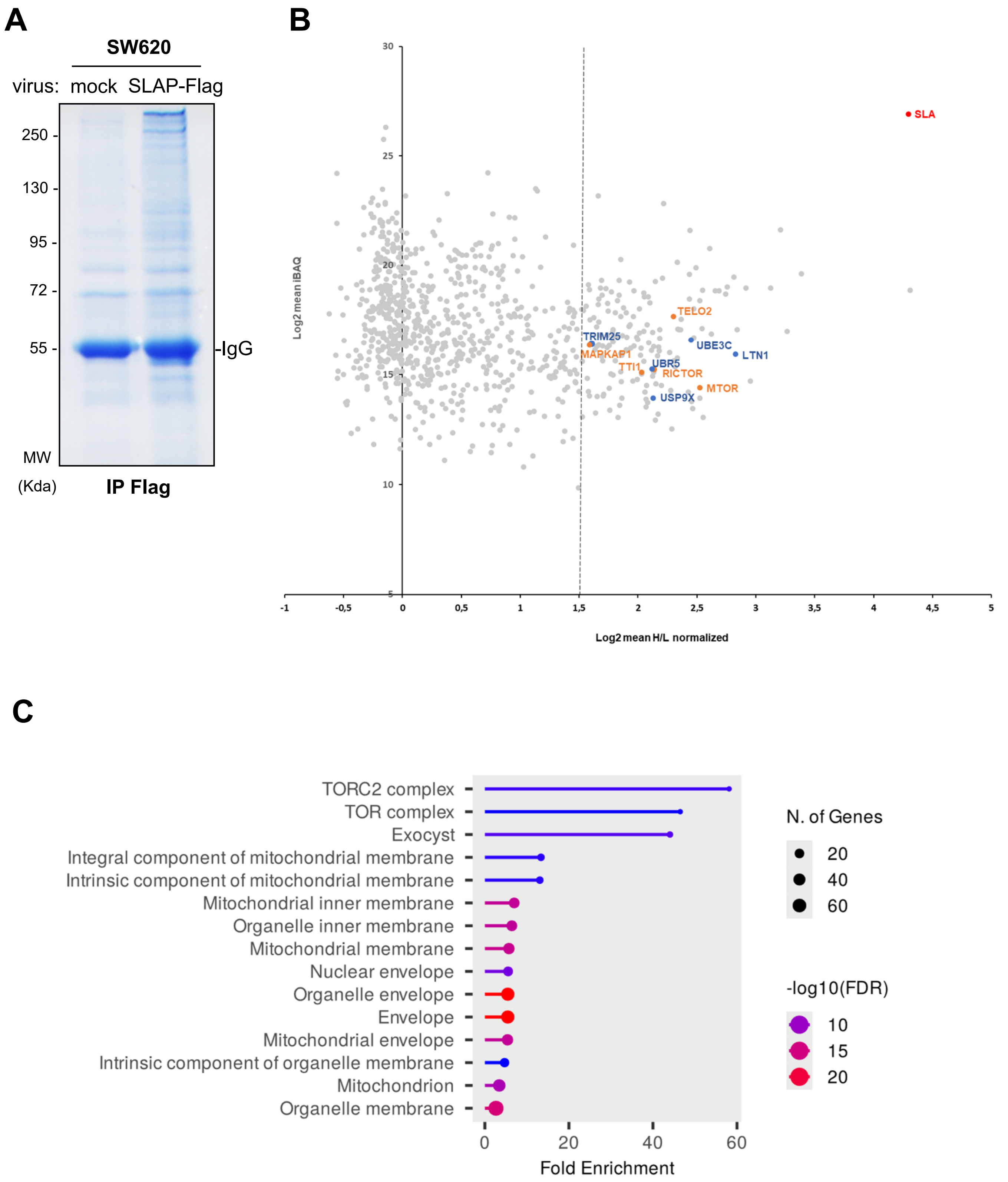

Figure S1: SILAC-based SLAP interactomics in SW620 cells. A: brillant blue staining of SDS-PAGE gel from SLAP-Flag immunoprecipitation in SW620 cells. **B:** graph showing the relative quantification (log2 mean intensity relative to the log2 mean H/L normalized ratio) of SLAP interactors (identified ≥2/3 independent experiments with ≥3 peptides). SLAP (red), mTORC2 components (orange) and ubiquitination factors (blue) are highlighted. **C:** GO analysis of SLAP interactors described in table S1.

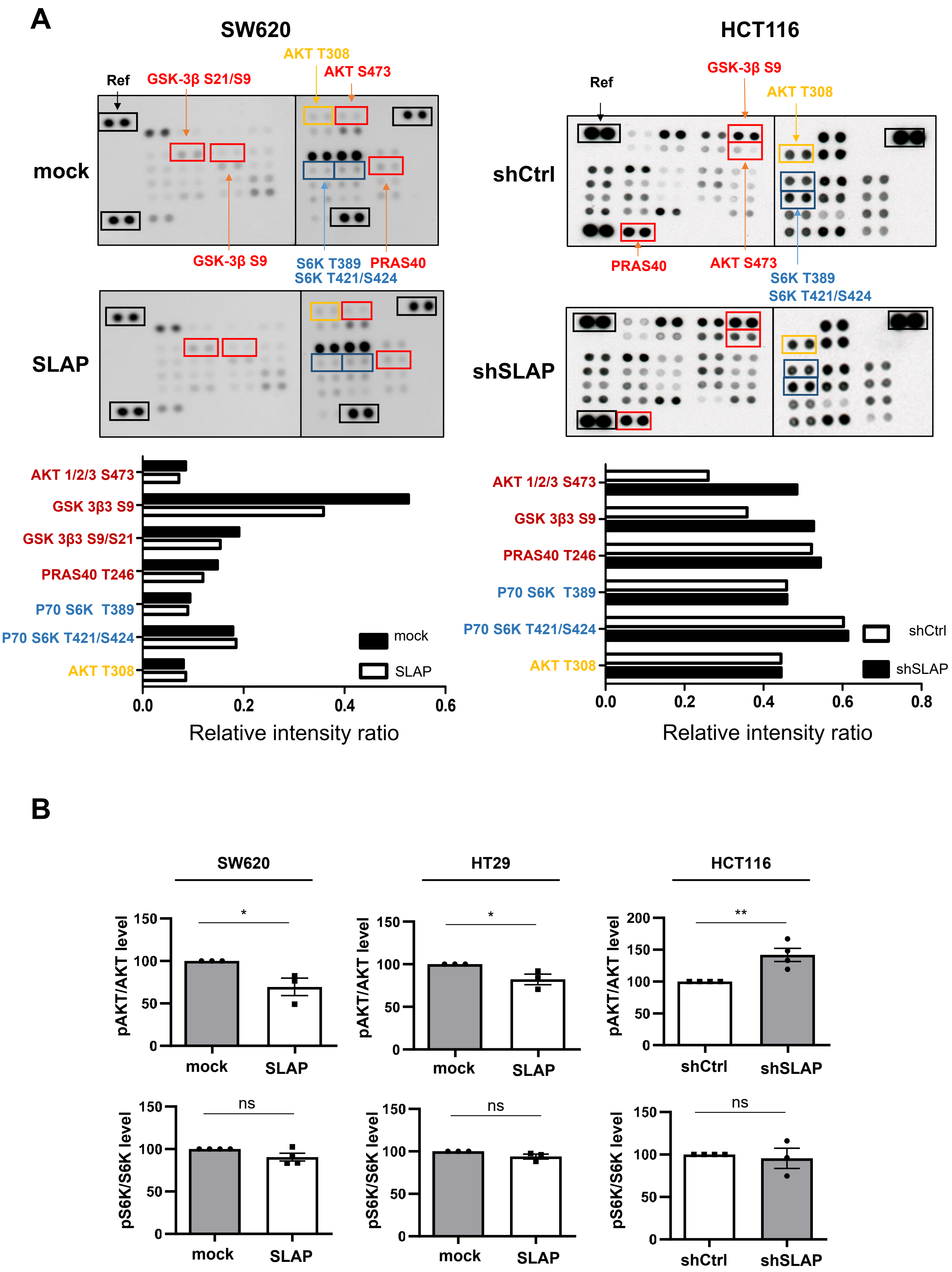
A
SW620
HCT116
mock
SLAP
shCtrl
shSLAP
Ref
GSK-3β S21/S9
AKT T308
AKT S473
GSK-3β S9
S6K T389
S6K T421/S424
PRAS40
AKT 1/2/3 S473
GSK 3β3 S9
GSK 3β3 S9/S21
PRAS40 T246
P70 S6K T389
P70 S6K T421/S424
AKT T308
Relative intensity ratio
B
HT29
pAKT/AKT level
pS6K/S6K level
*
**
ns

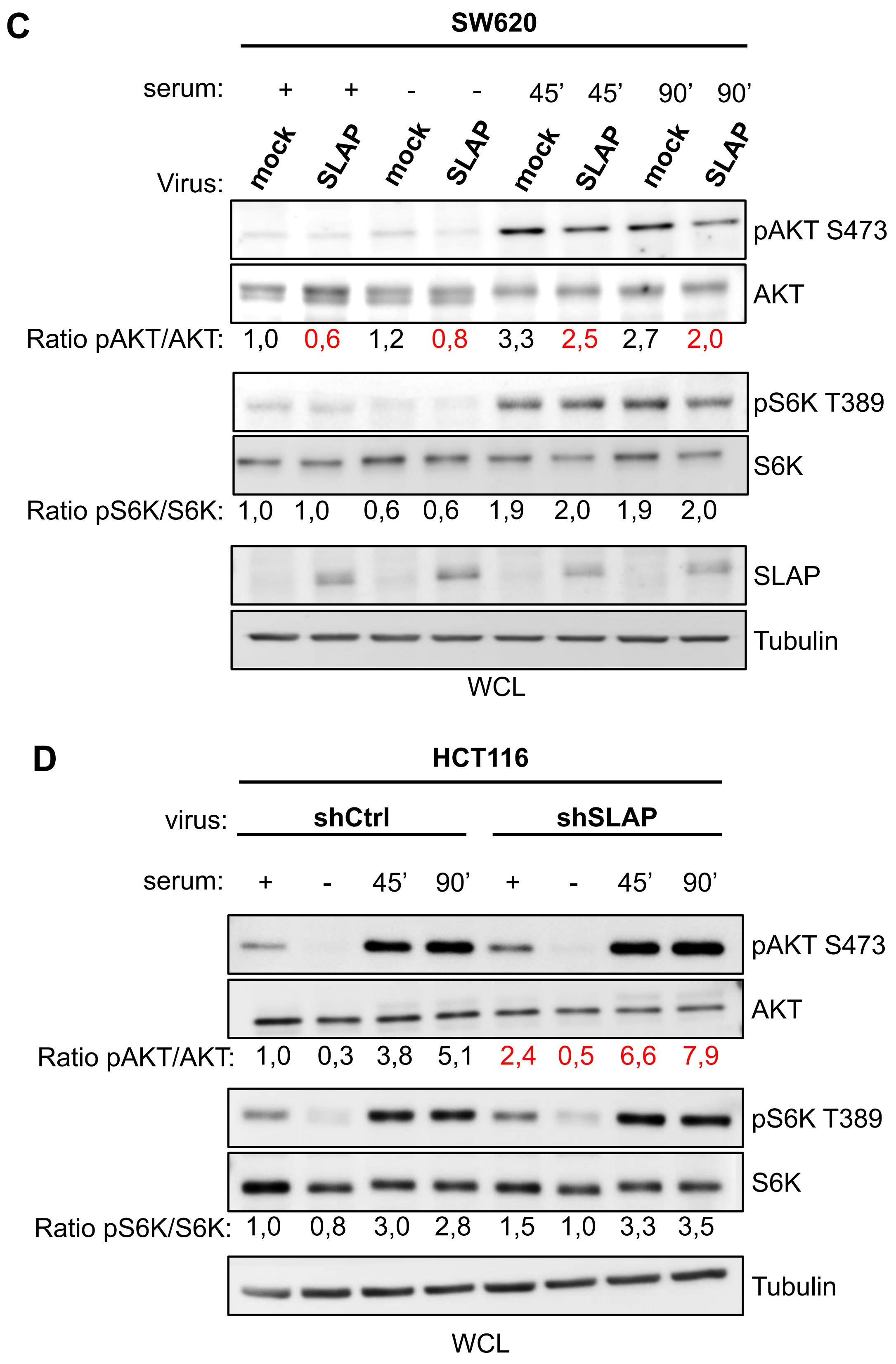

Figure S2: SLAP regulates mTORC2 phospho-signaling in CRC cells. **A**: phospho-kinase array of indicated cell-lysates; **B**: quantification of SLAP-dependent phosphorylation of main mTORC1/2 substrates in indicated CRC cells (mean ± SEM, n=3-4, ns: $p>0.05$, *$p\leq0.05$, **$p\leq0.01$; t-test). **C** (SW620) and **D** (HCT116): WB analysis of selected mTOR substrates phosphorylation in quiescent CRC cells expressing or not SLAP that were stimulated with 10% serum at indicated time points. The relative phosphorylation level is shown (ratio).

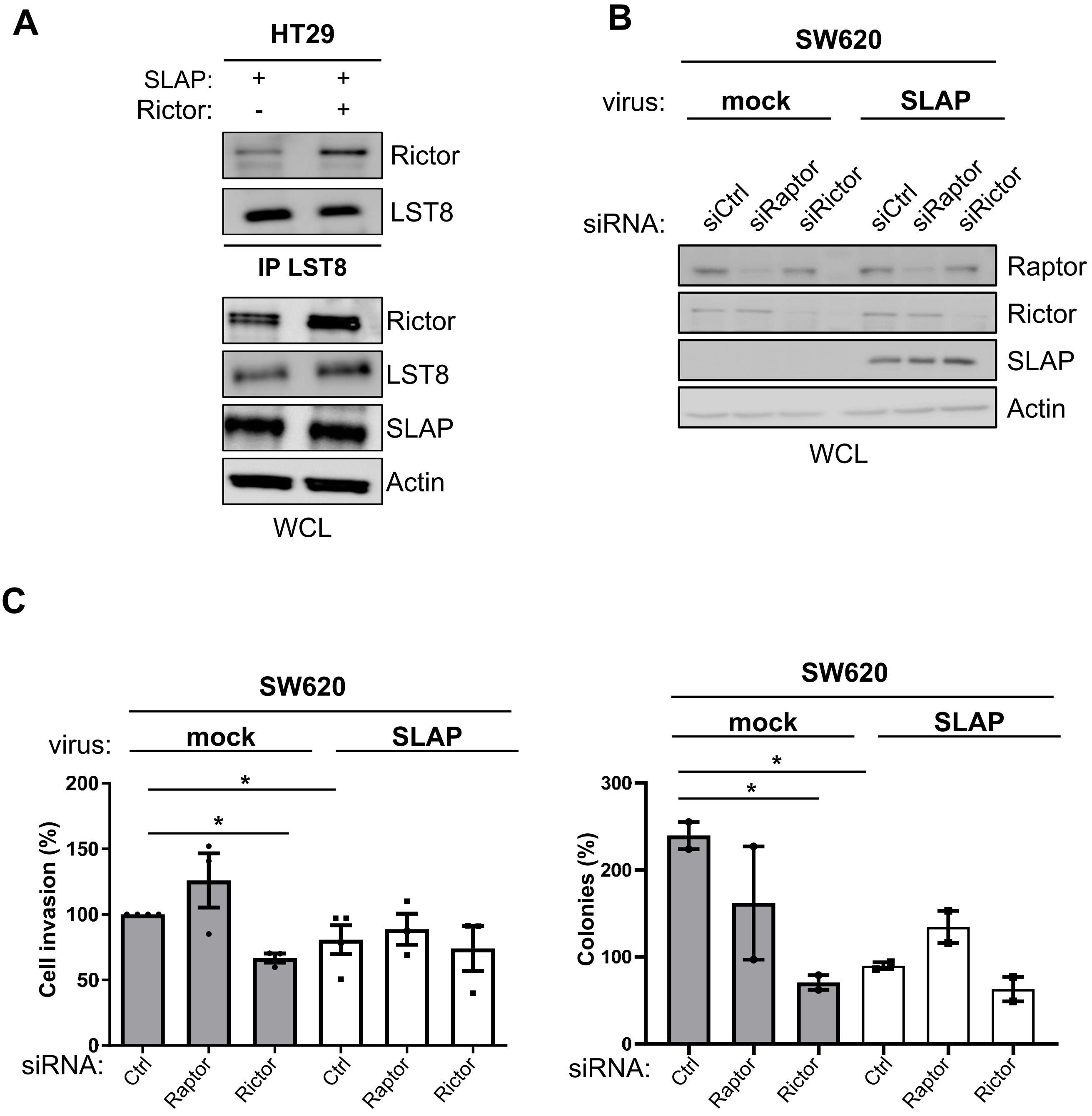

Figure S3: SLAP-dependent mTORC2 transforming function in CRC cells. **A**: Rictor overexpression enhances mTORC2 complex formation in HT29 cells overexpressing SLAP. **B-C**: Rictor-dependent cell growth in soft agar and cell invasion in matrigel of SW620 cells expressing or not SLAP. **B** :WB of indicated protein levels. **C**: Rictor-dependent SLAP anti-oncogenic effect in SW620. Mean ± SEM, n=4-6; *p<0.05; **p<0.01 Unpaired t-test.

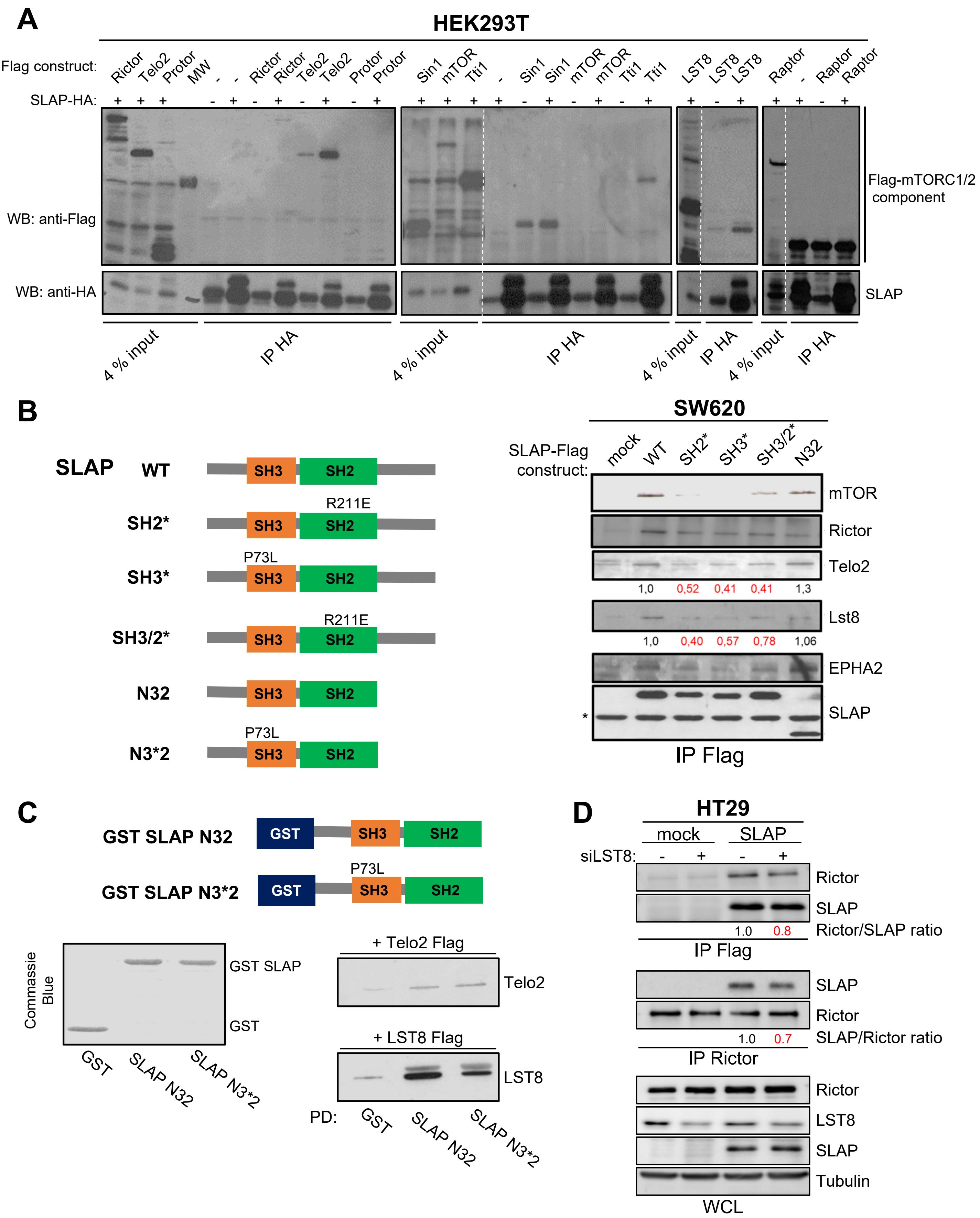

Figure S4: SLAP interaction with TELO2 and LST8. **A**: SLAP co-immunoprecipitation with mTORC1 and 2 components that were expressed in HEK293T cells. **B**: Mutagenesis analysis of SLAP interaction with mTORC2 components in SW620 cells transduced with indicated SLAP-Flag constructs. EPHA2 was used as a positive control **C**: GST-SLAP pull-down (PD) using HEK293T cell-lysates transfected with TELO2 and LST8 constructs as shown. **D**: SLAP-Rictor interaction is mediated by mLST8 expression in HT29 cells. *: Lc of ip Flag antibody. Dotted lines in A indicate where the image was cropped to remove irrelevant lanes. Raw data for the full image are provided in the Source Data file.

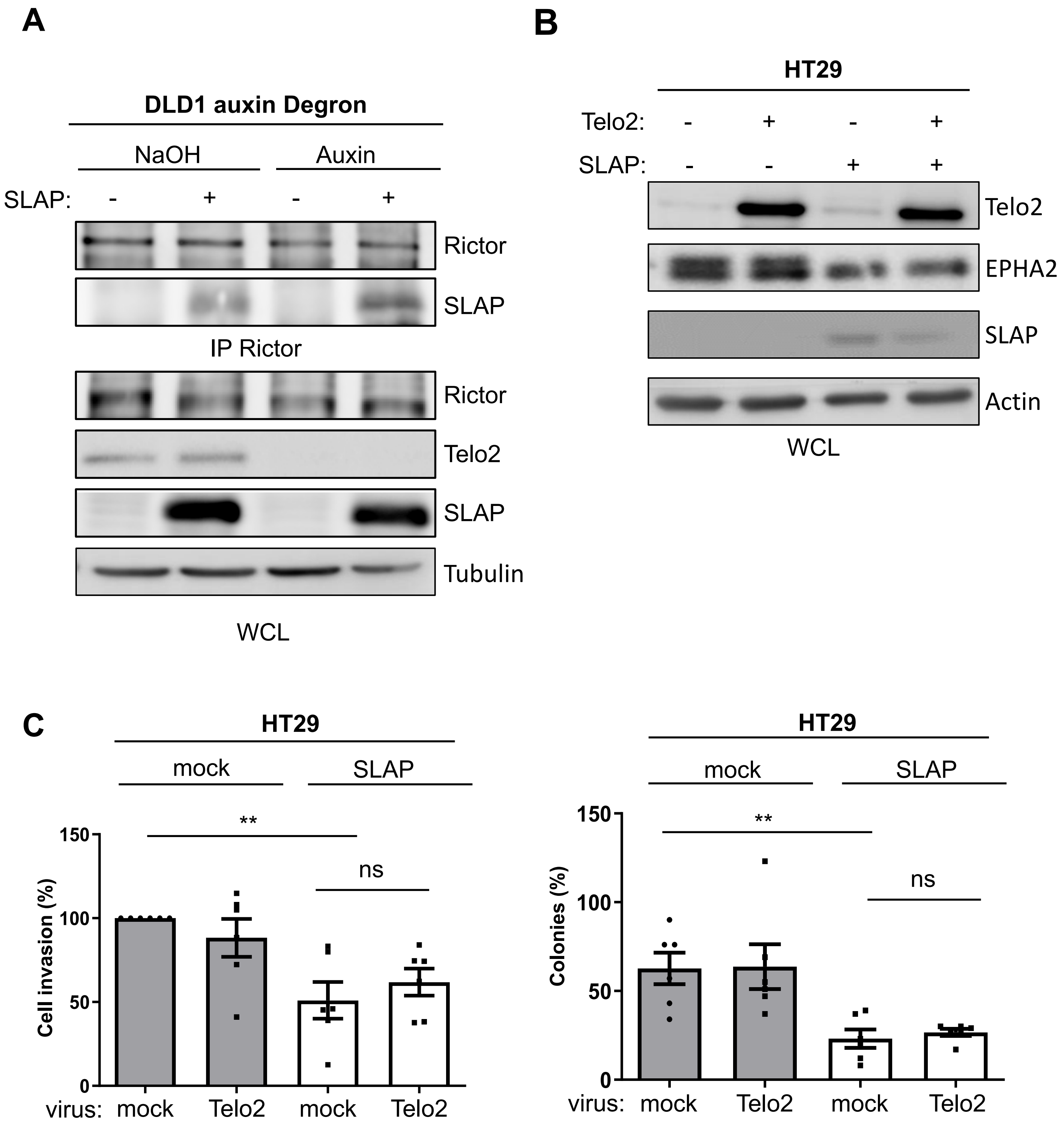

Figure S5: Telo2 is dispensable for SLAP anti-oncogenic effect. A: Telo2 degradation via a auxin-induced degron system does not affect SLAP association with Rictor in DLD1 CRC cells. **B:** WB of Telo2 overexpression in control and SLAP overexpressing HT29 cells. The level of the SLAP target EPHA2 is shown. **C**: Telo2 overexpression does not overcome SLAP anti-oncogenic properties in HT29 cells. The Mean ± SEM, n=3-5; ns $p>0.05$; *$p<0.05$; **$p<0.01$; Unpaired t-test.

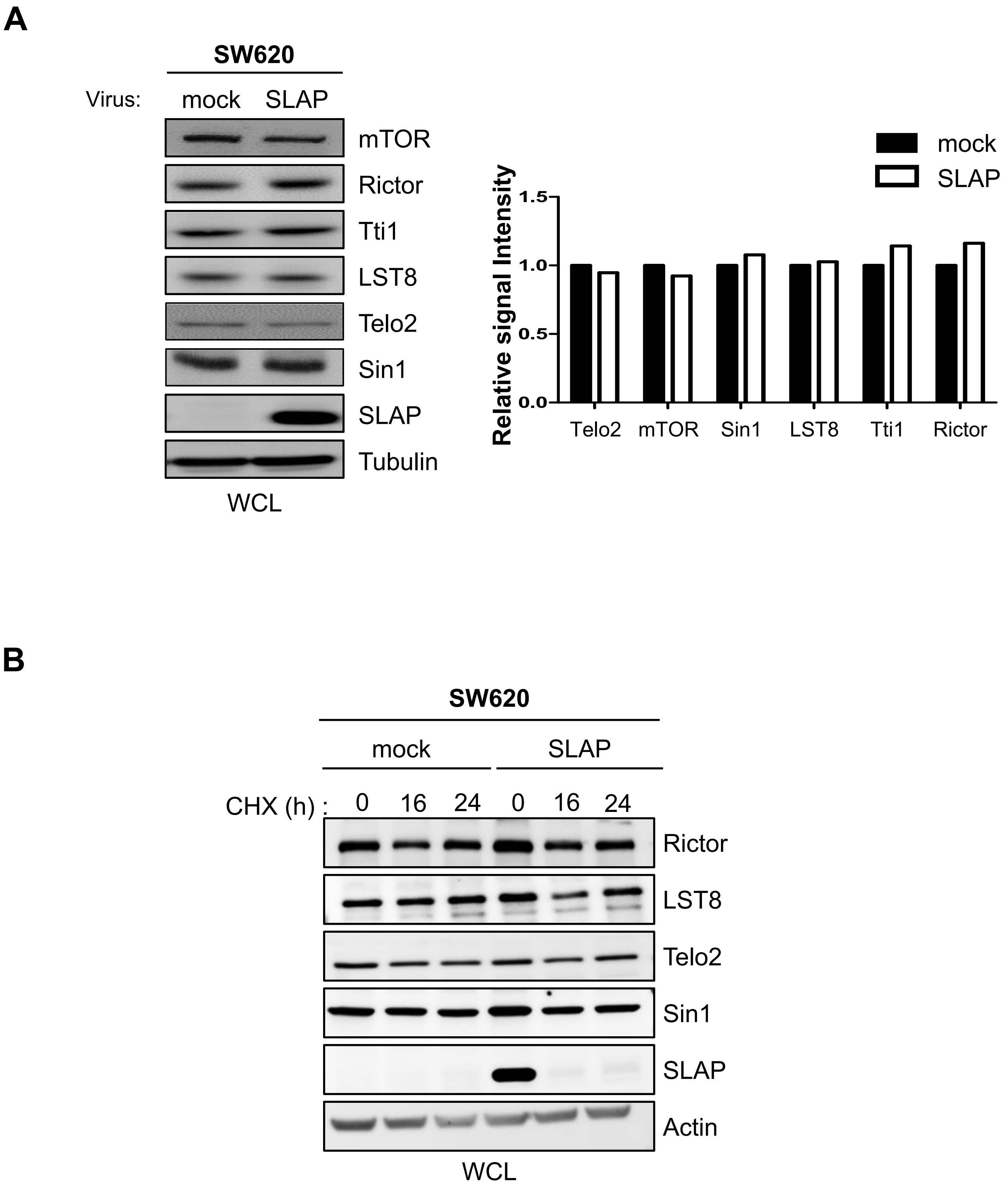

Figure S6: SLAP does not affect the protein stability of mTORC2 components. A: SLAP does not affect mTORC2 proteins levels in CRC cells. Is shown a representative example (left) and quantification (mean, n=2) (right). **B:** SLAP does not affect mTORC2 protein turnover, as shown from cell treatment with cyclohexymide at indicated times (CHX; 100µg/ml).

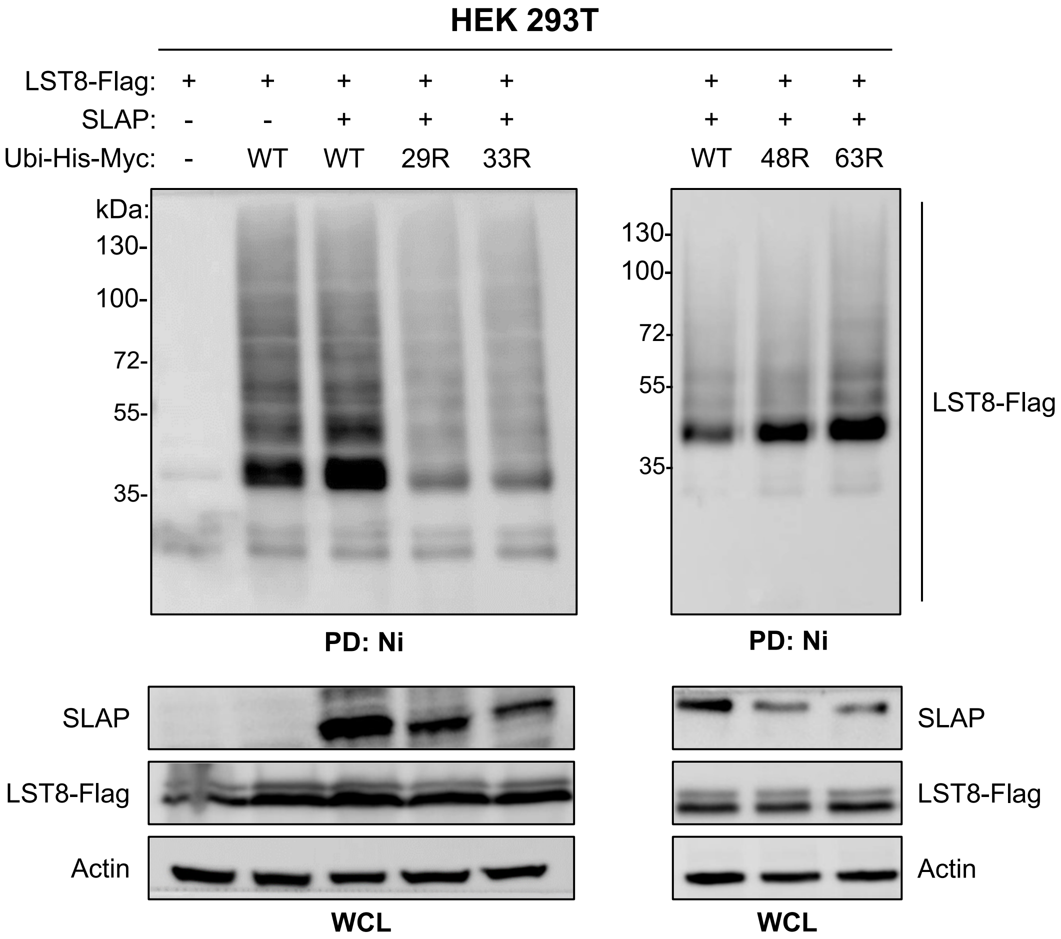

Figure S7: SLAP-mediated LST8 ubiquitination involves branching on Ubiquitin K29 and K33. SLAP-dependent LST8 ubiquitination assay in HEK 293T cells using SLAP WT or indicated KR Ubiquitin (Ub-His-Myc) mutants.

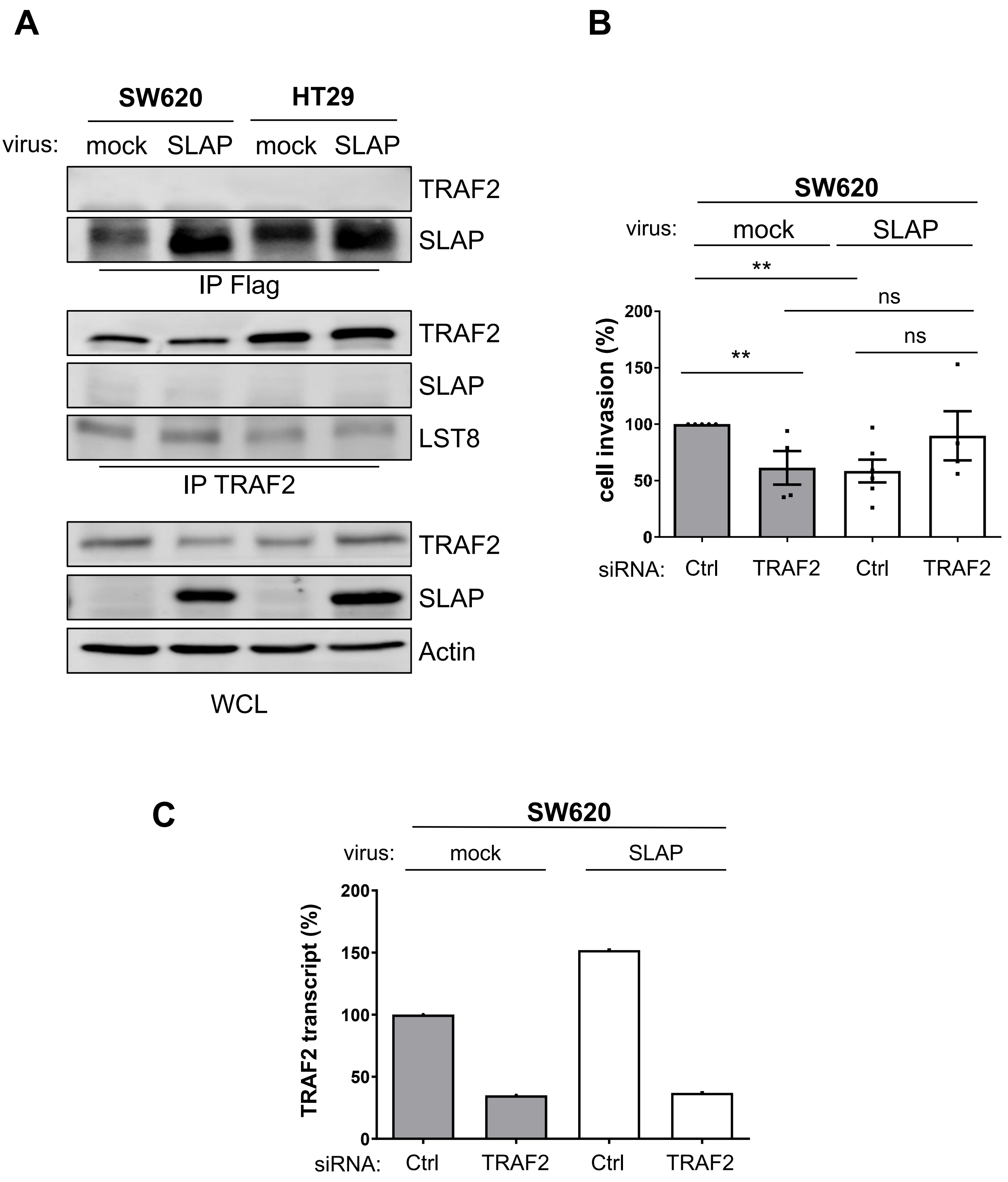

Figure S8: TRAF2 is not involved in SLAP signaling. A: SLAP does not interact with TRAF2 in CRC cells. **B.** TRAF2 depletion does not affect SLAP anti-invasive properties in CRC cells. Is shown the mean ± SEM, n=5; ns $p>0.05$; *$p<0.05$, **$p<0.01$; Unpaired t-test. **C.** siRNA silencing of TRAF2 expression by qPCR.

A

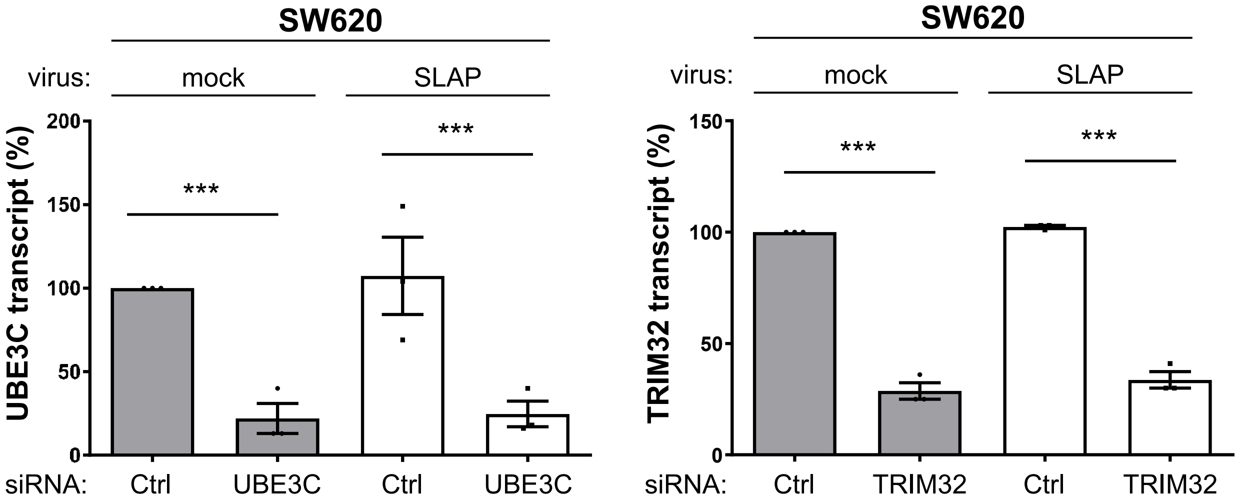

B

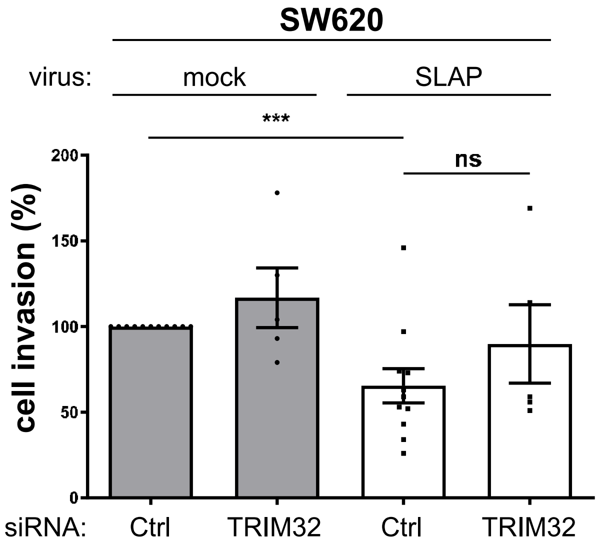

Figure S9: UBE3C is implicated in SLAP signaling. A: qPCR analysis of siRNA UBE3C (left) and TRIM32 (right) silencing (n=3). **B**: TRIM32 depletion does not significantly affect SLAP anti-invasive properties (mean ± SEM, n=5); ns $p>0.05$; ***$p<0.001$; Unpaired t-test.

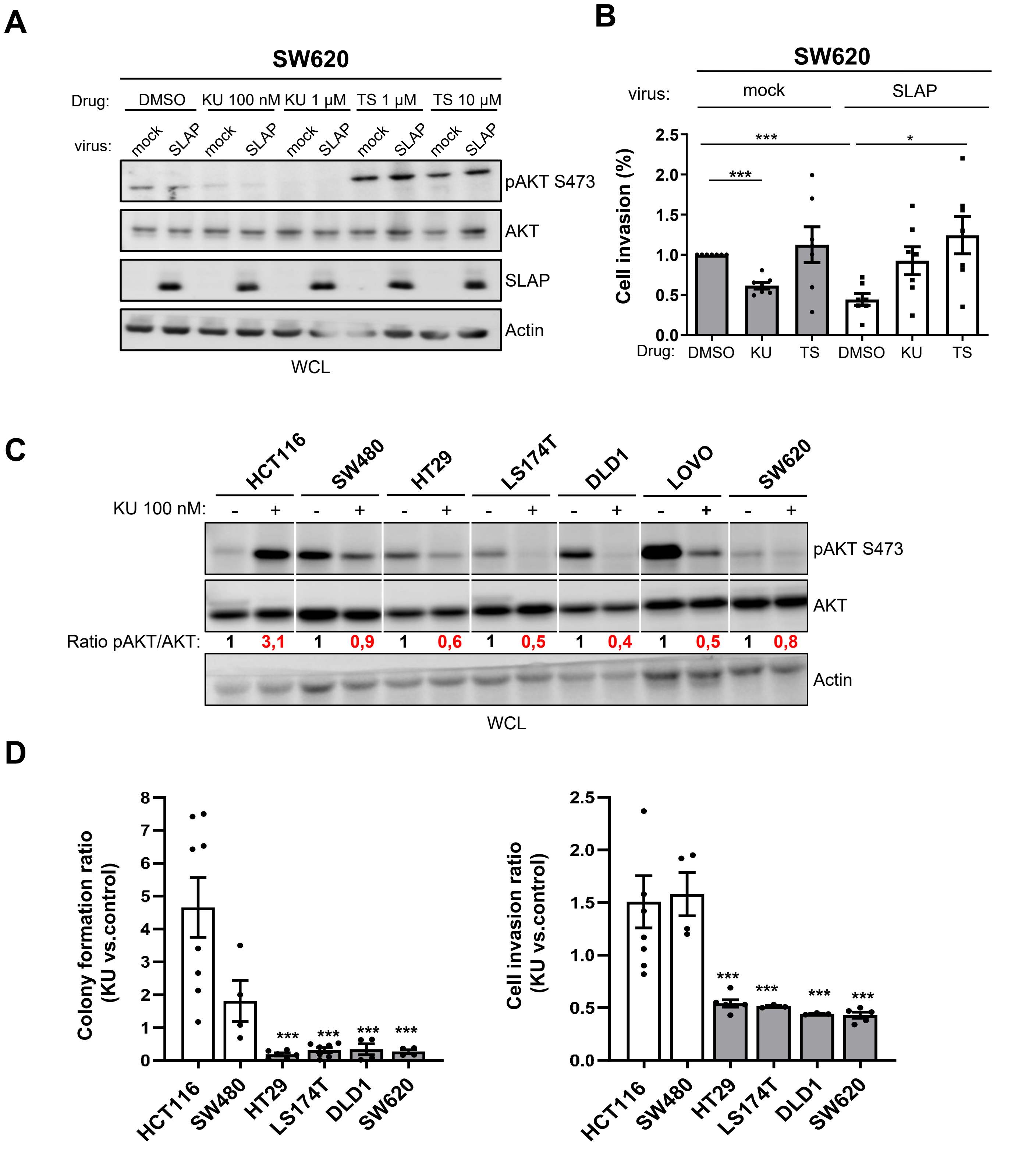

Figure S10. SLAP overexpression reduces mTORCi activity in SW620 cells. A: pAKT levels and **(B)** cell invasion in SW620 cells treated with indicated mTORCinhibitors. **C–D:** Correlation between endogenous SLAP expression and sensitivity to KU (100 nM) across CRC cell lines: **C**: relative pAKT/AKT level in response to KU for each cell line, **D**: cell transformation. Cell lines were grouped by SLAP expression: SLAP-high (white) and SLAP-low (grey) (Naudin et al, Nat Commun 2014:5:3159). SLAP-low lines showed reduction in colony formation and invasion (i.e. ratio <1), in contrast to SLAP-high lines (i.e. ratio >1). Data represent mean ± SEM ($n \geq 3$, ***$p < 0.001$, unpaired t-test).

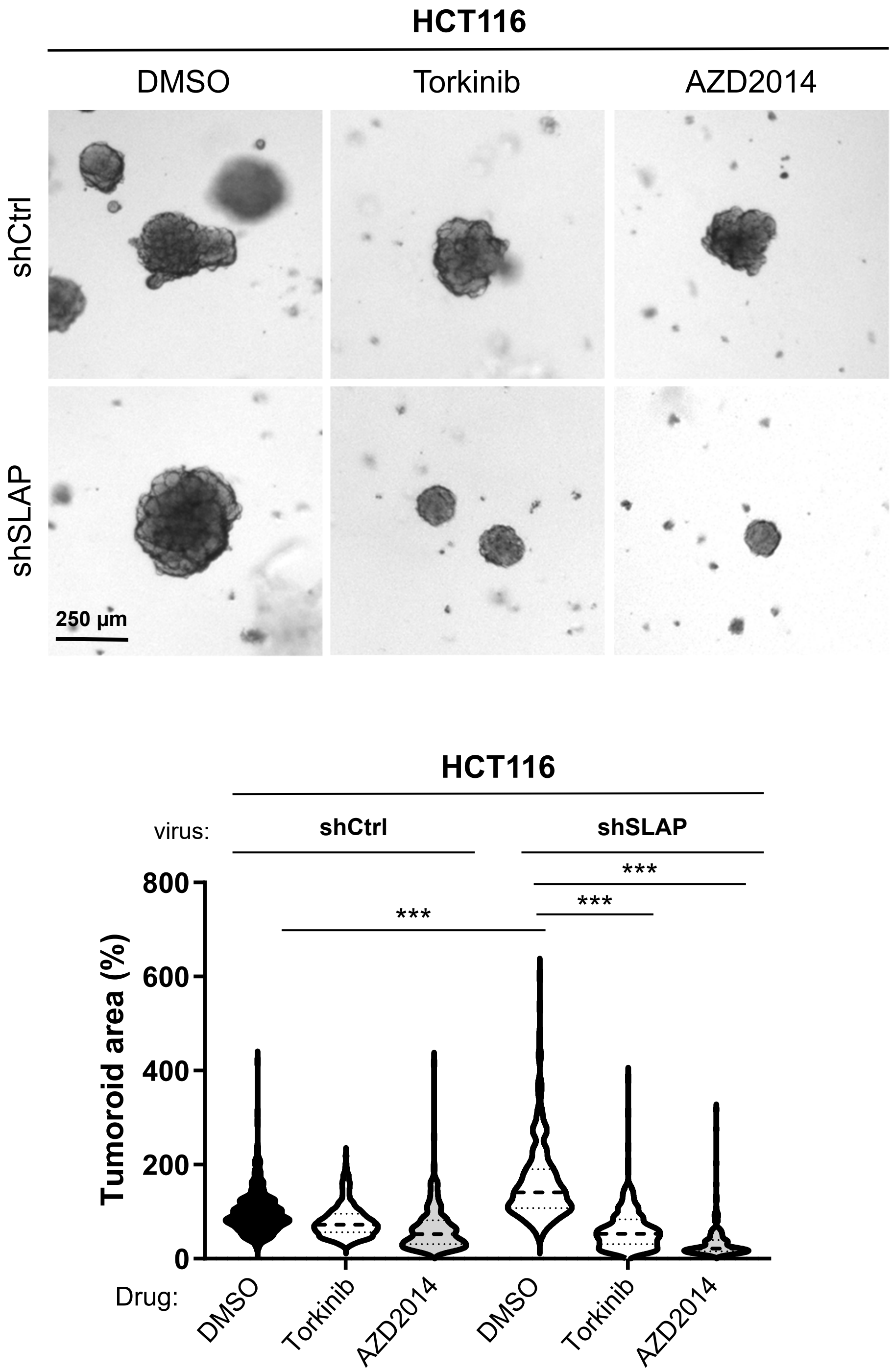

Figure S11: SLAP depletion enhances the mTORCi activity in tumoroids derived from HCT116 cells. A representative example (top) and its quantification (bottom) of indicated mTORCi (Torkinib/ PP242, AZD2014) effects on tumoroid growth. Is shown a violin representation of the mean, 80-100 tumoroids analyzed/replicate, n=3; ***p<0.001; Unpaired t-test.

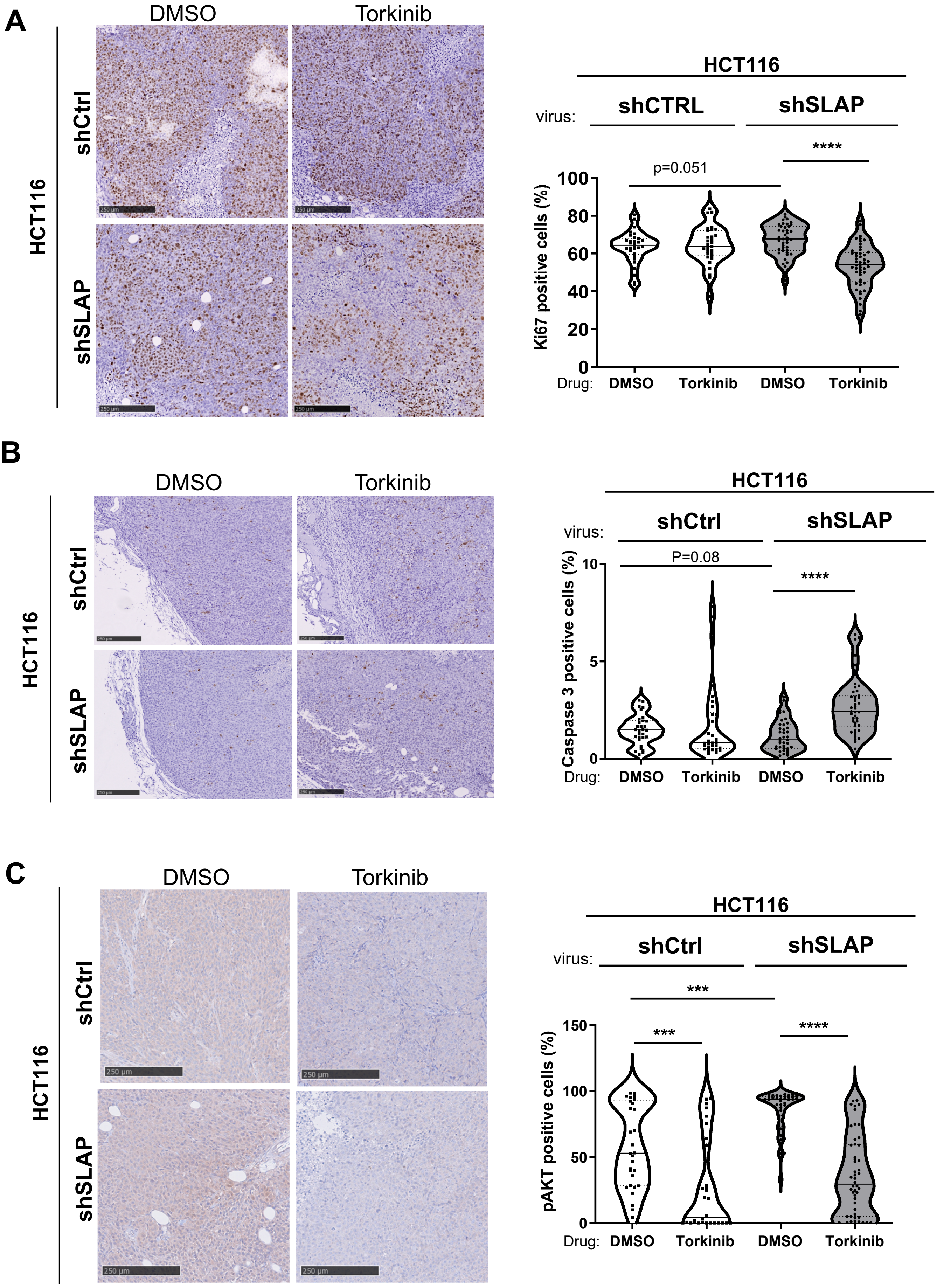

Figure S12: SLAP depletion sensistizes CRC cells reponse to mTORCi *in vivo*. IHC analysis of (**A**) cell proliferation (Ki67), (**B**) apoptosis (Caspase 3) and (**C**) pS473 AKT level in indicated tumors treated or not with Torkinib (mean ± SEM, n=5 mice; p close to 0.05 is indicated; ***p<0.005, ****p<0.001; Mann-Withney t-test).
