# Supplementary material for: SLAP controls mTORC2 integrity via UBE3C-mediated non-degradative mLST8 ubiquitination to suppress colorectal tumorigenesis
Source: Cell Death Differ. 2025 Dec 15;33(5):1020–35. doi: 10.1038/s41418-025-01633-1 (PMC13156305; doi:10.1038/s41418-025-01633-1)

Fig. 1A

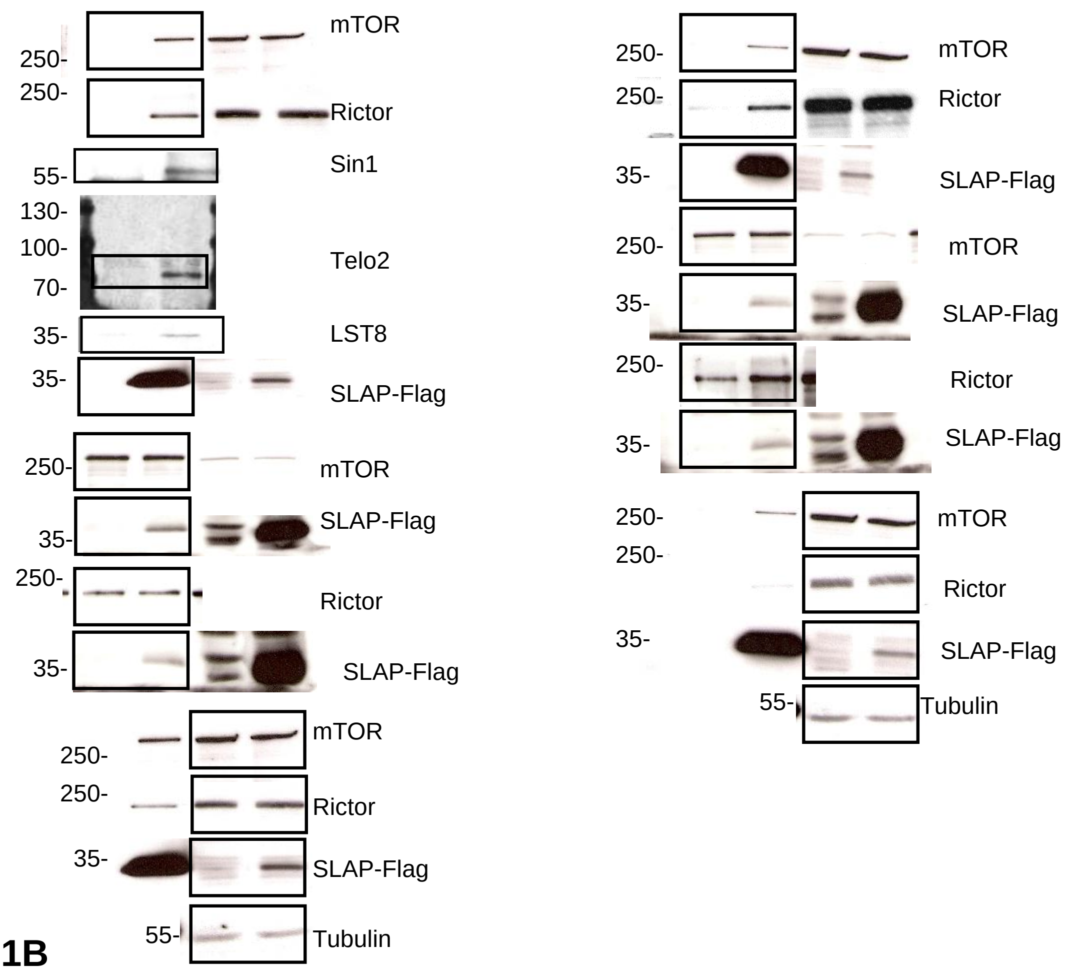
mTOR
250-
250-
Rictor
55-
Sin1
130-
100-
70-
Telo2
35-
LST8
35-
SLAP-Flag
250-
mTOR
SLAP-Flag
35-
250-
Rictor
35-
SLAP-Flag
mTOR
250-
250-
Rictor
35-
SLAP-Flag
55-
Tubulin
250-
mTOR
250-
Rictor
35-
SLAP-Flag
250-
mTOR
35-
SLAP-Flag
250-
Rictor
35-
SLAP-Flag
250-
mTOR
250-
Rictor
35-
SLAP-Flag
55-
Tubulin

Fig. 1B

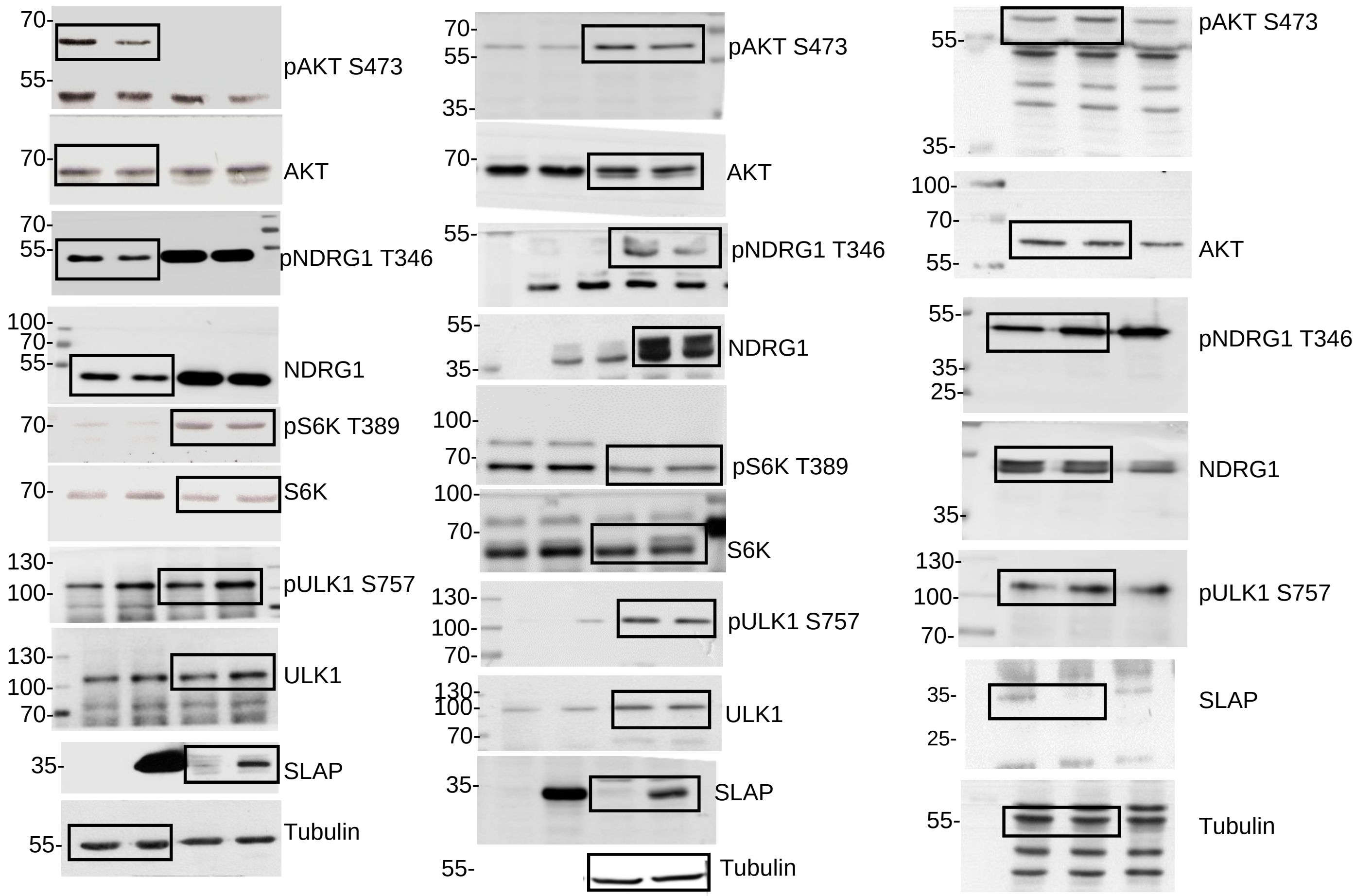
70-
55-
pAKT S473
70-
AKT
70-
55-
pNDRG1 T346
100-
70-
55-
NDRG1
70-
pS6K T389
70-
S6K
130-
100-
pULK1 S757
130-
100-
70-
ULK1
35-
SLAP
55-
Tubulin
70-
55-
35-
pAKT S473
70-
AKT
55-
pNDRG1 T346
55-
35-
NDRG1
100-
70-
pS6K T389
100-
70-
S6K
130-
100-
70-
pULK1 S757
130-
100-
70-
ULK1
35-
SLAP
55-
Tubulin
55-
35-
pAKT S473
100-
70-
55-
AKT
55-
35-
25-
pNDRG1 T346
NDRG1
35-
130-
100-
70-
pULK1 S757
35-
25-
SLAP
55-
Tubulin

Fig. 1B

Fig. 2A

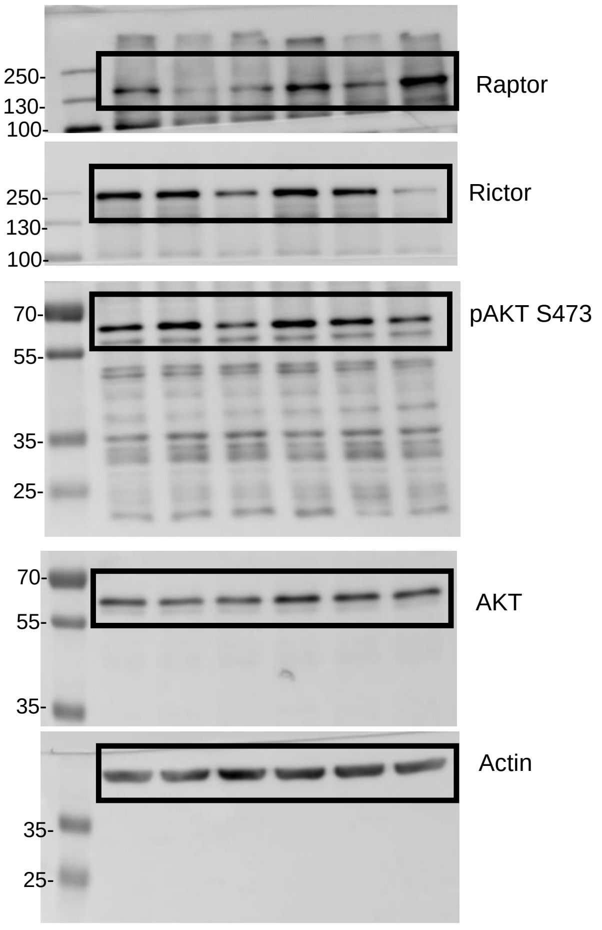

Fig. 2B

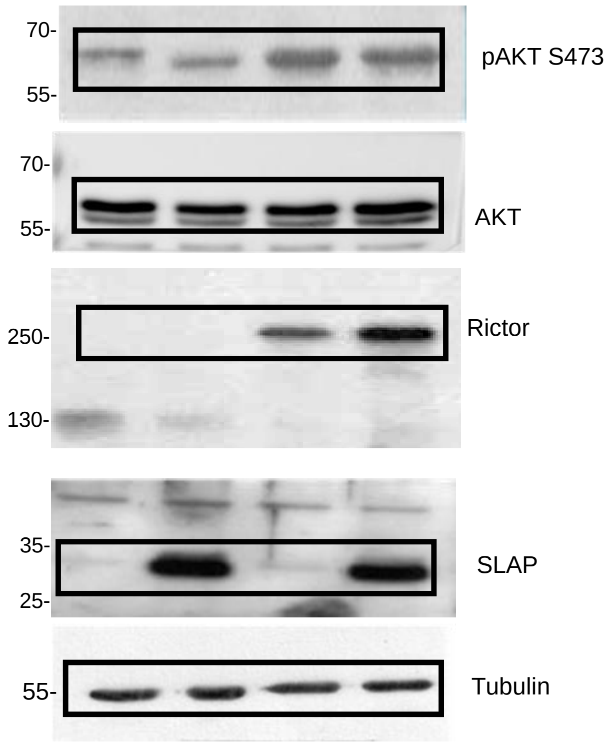

Fig. 3A

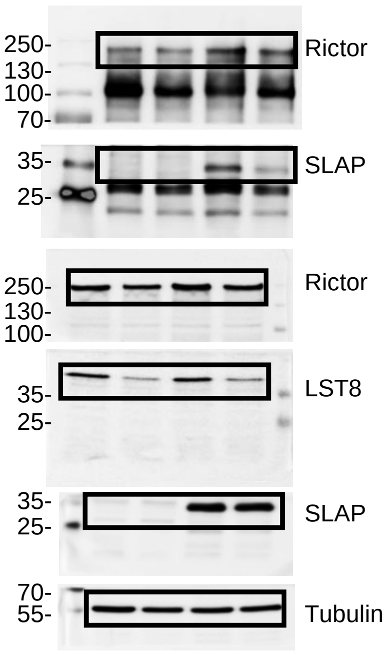

Fig. 3B

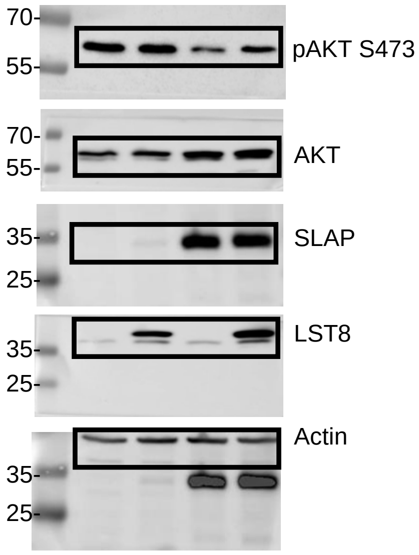

Fig. 3C

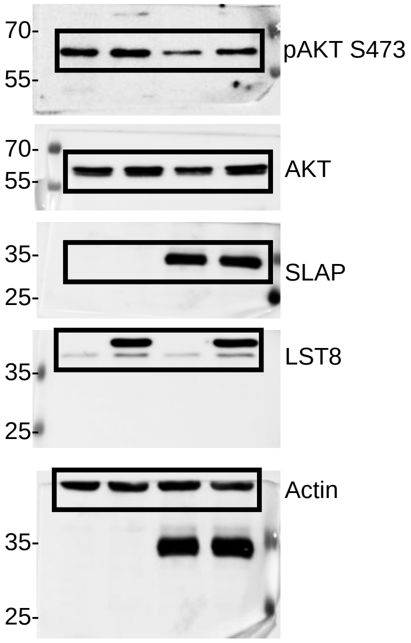

Fig. 3D

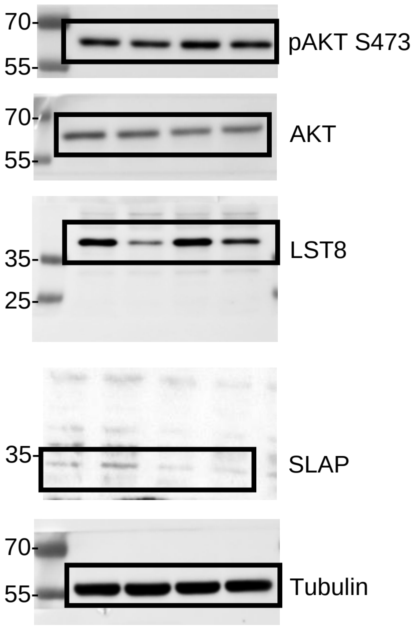

Fig. 4A

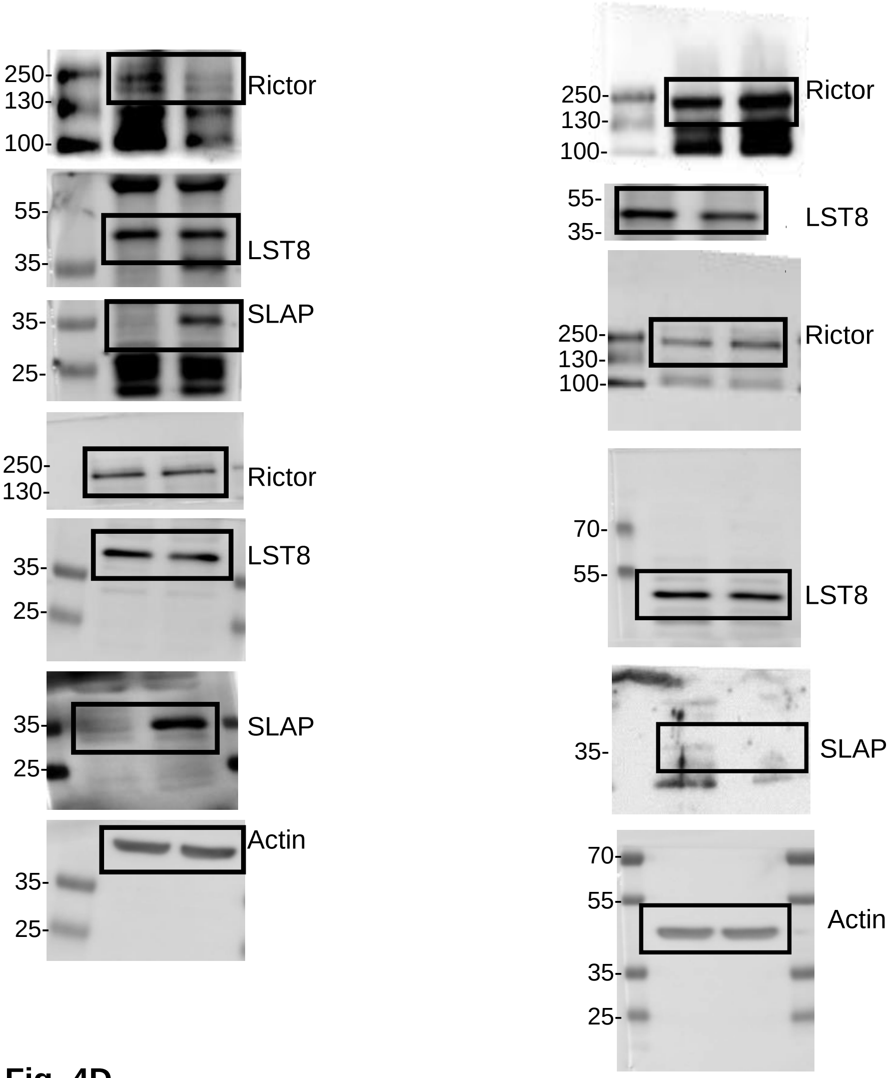
250-
130-
100-
Rictor
55-
35-
LST8
35-
25-
SLAP
250-
130-
Rictor
35-
25-
LST8
35-
25-
SLAP
35-
25-
Actin
250-
130-
100-
Rictor
55-
35-
LST8
250-
130-
100-
Rictor
70-
55-
LST8
35-
SLAP
70-
55-
35-
25-
Actin

Fig. 4D

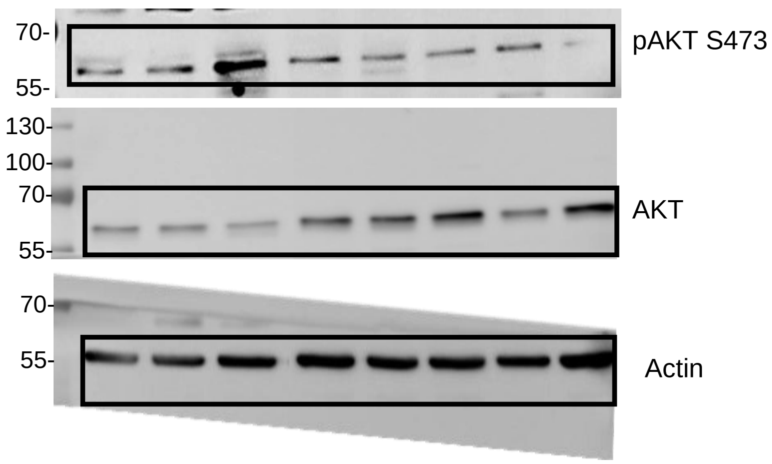
70-
55-
pAKT S473
130-
100-
70-
55-
AKT
70-
55-
Actin

Fig. 5 A

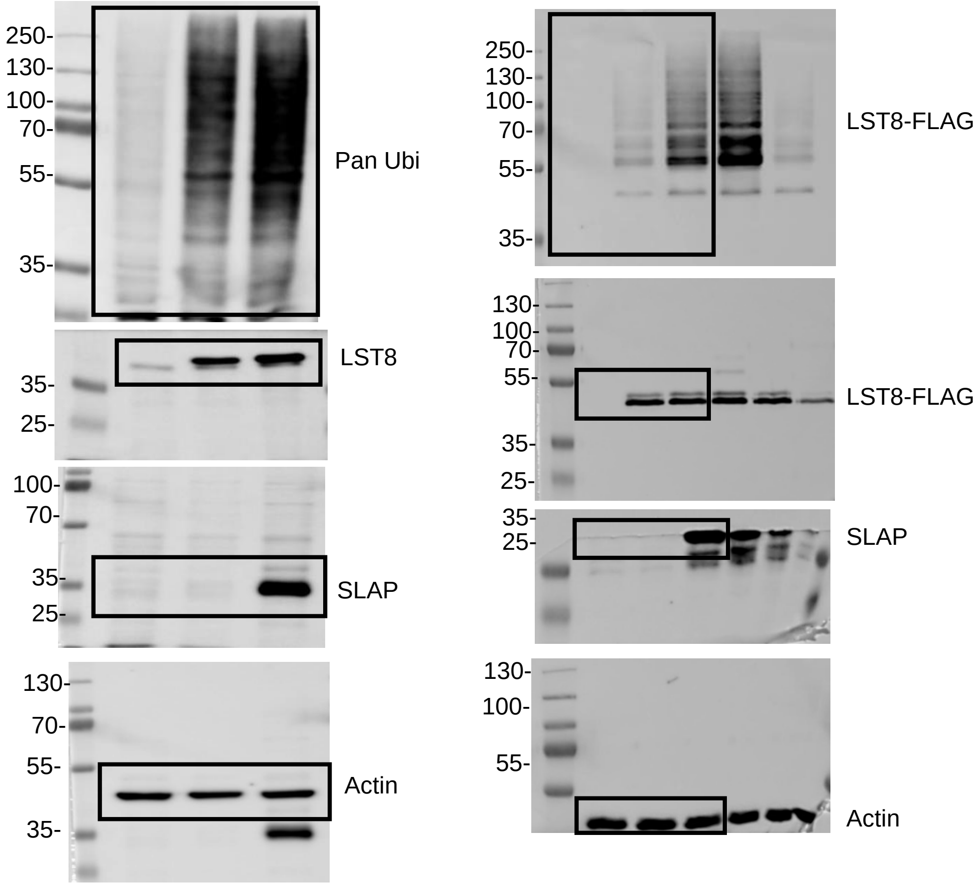

Fig. 5B

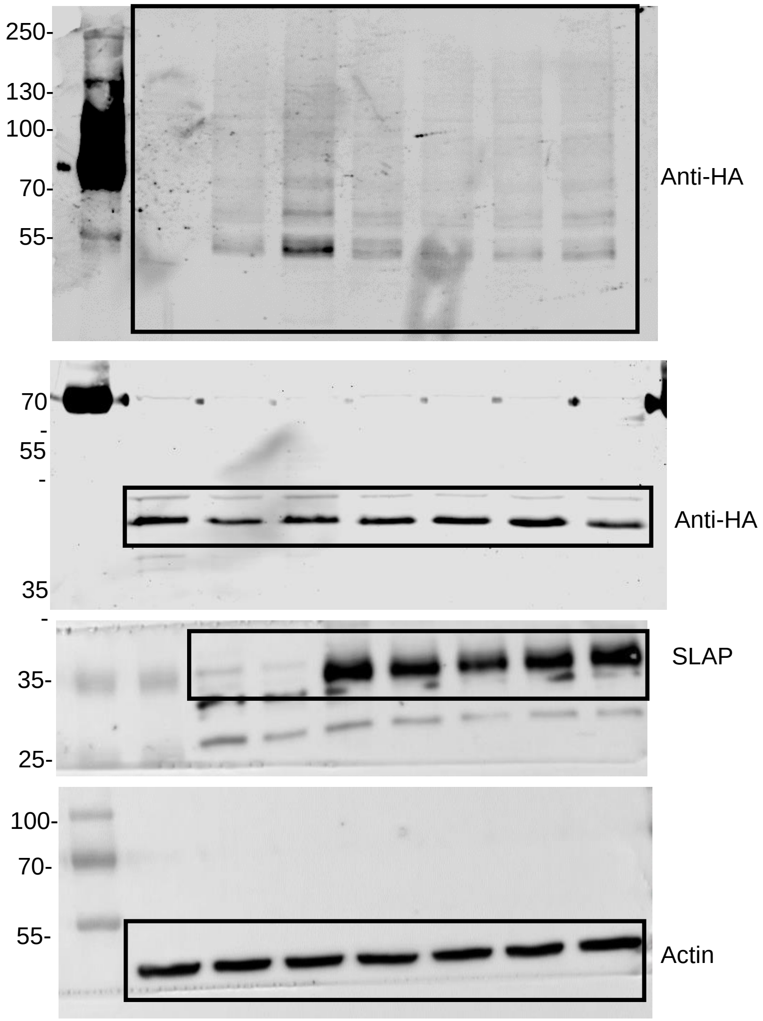

Fig. 5C

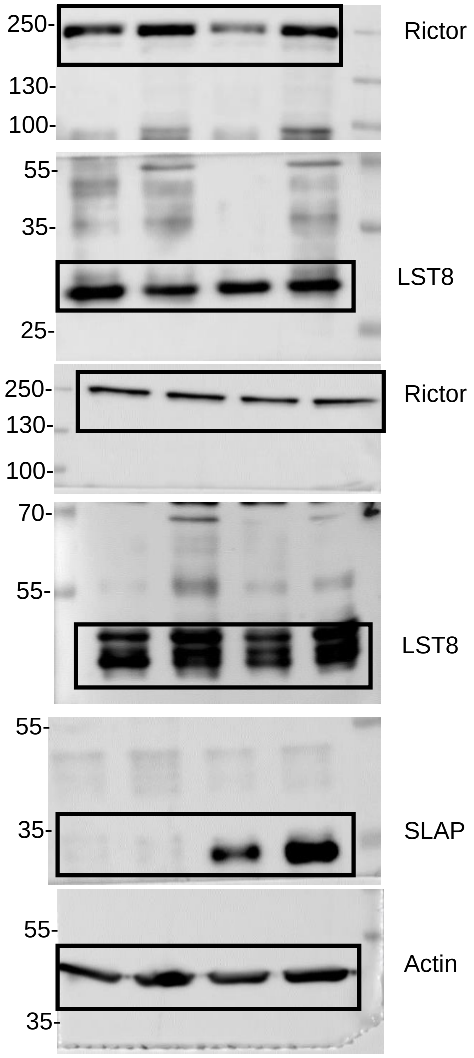

Fig. 6 A

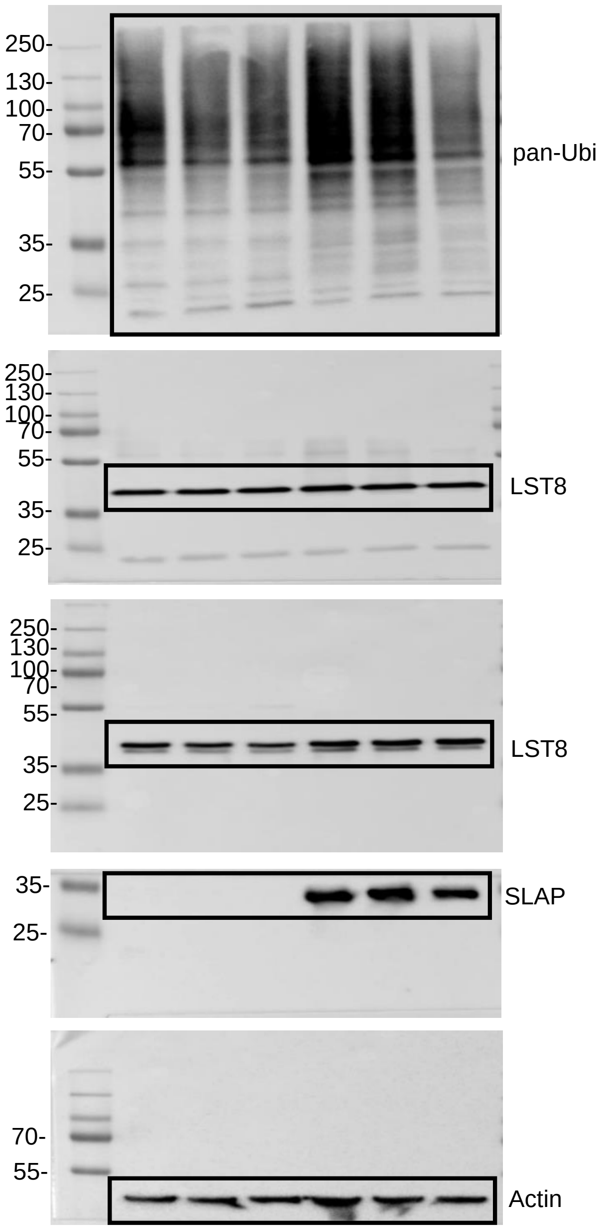
250-
130-
100-
70-
55-
35-
25-
pan-Ubi
250-
130-
100-
70-
55-
35-
25-
LST8
250-
130-
100-
70-
55-
35-
25-
LST8
35-
25-
SLAP
70-
55-
Actin

Fig. 6 B

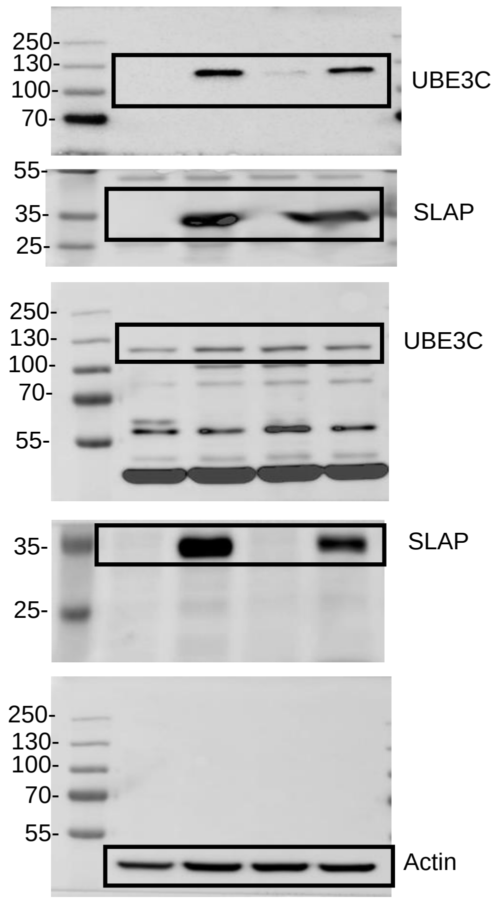
250-
130-
100-
70-
UBE3C
55-
35-
25-
SLAP
250-
130-
100-
70-
55-
UBE3C
35-
25-
SLAP
250-
130-
100-
70-
55-
Actin

Fig. 6 C

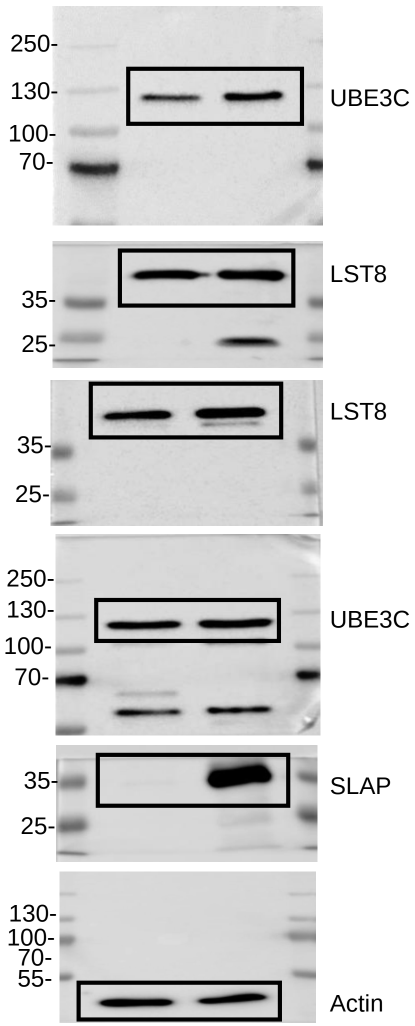
250-
130-
100-
70-
UBE3C
35-
25-
LST8
35-
25-
LST8
250-
130-
100-
70-
UBE3C
35-
25-
SLAP
130-
100-
70-
55-
Actin

Fig. 6 D

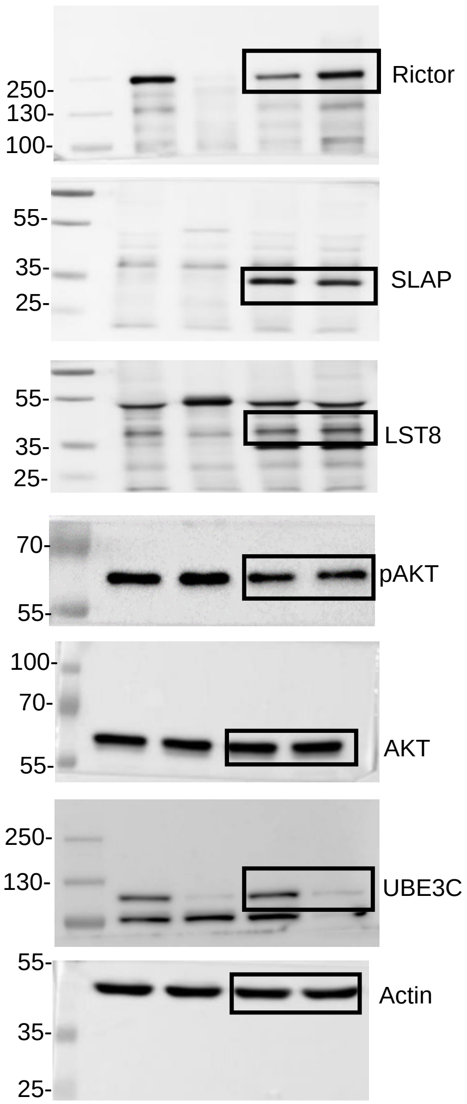
250-
130-
100-
Rictor
55-
35-
25-
SLAP
55-
35-
25-
LST8
70-
55-
pAKT
100-
70-
55-
AKT
250-
130-
UBE3C
55-
35-
25-
Actin

Fig. 7A

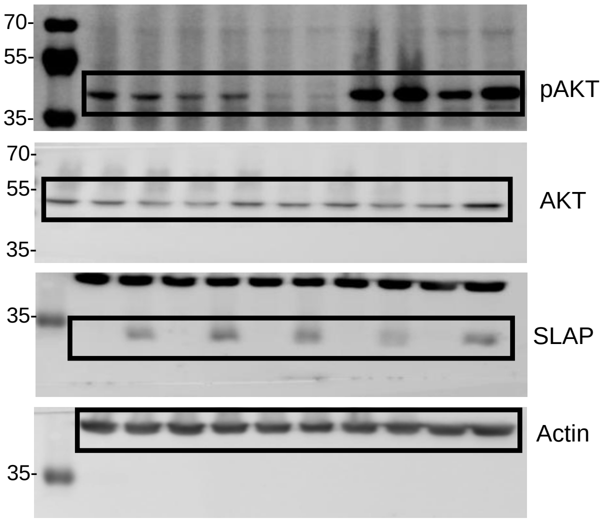

Fig. 7B

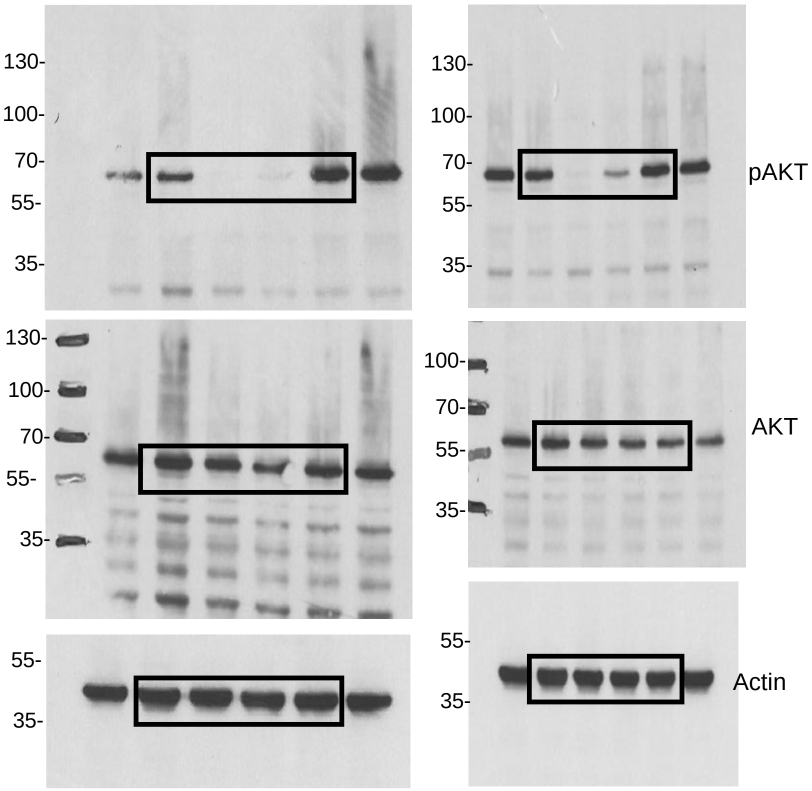

Fig. 8C

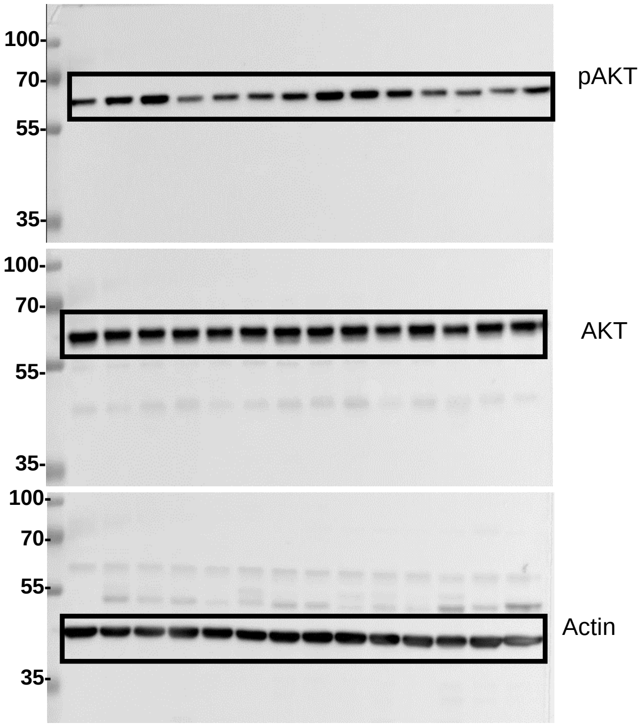

Fig. S1A

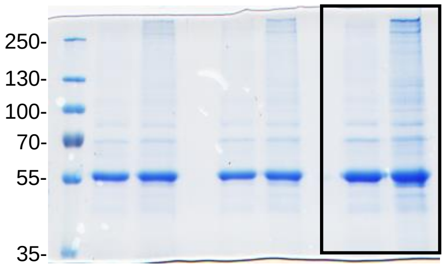

Fig. S2A

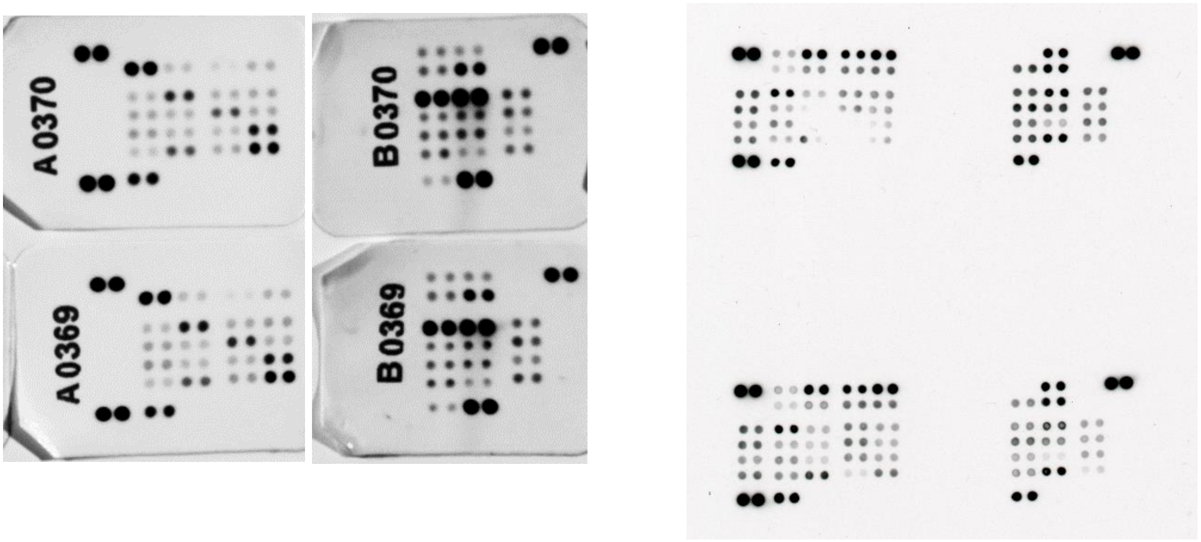

Fig. S2C

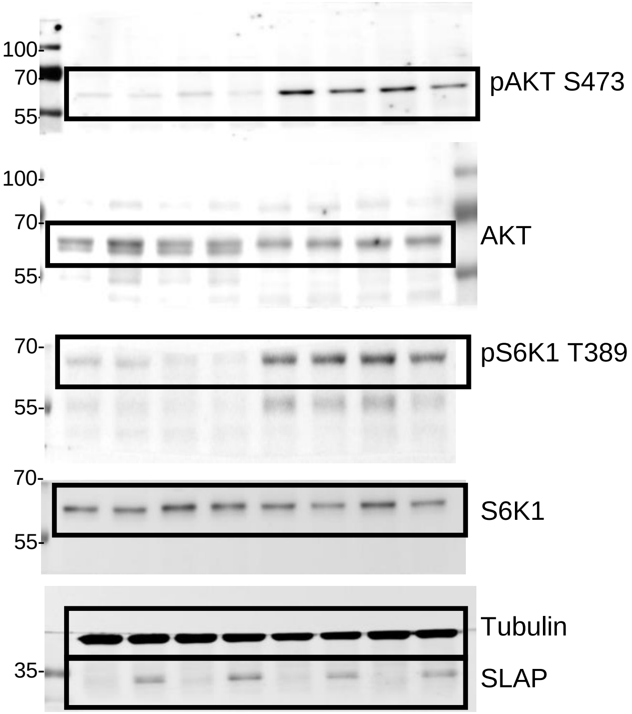

Fig. S2D

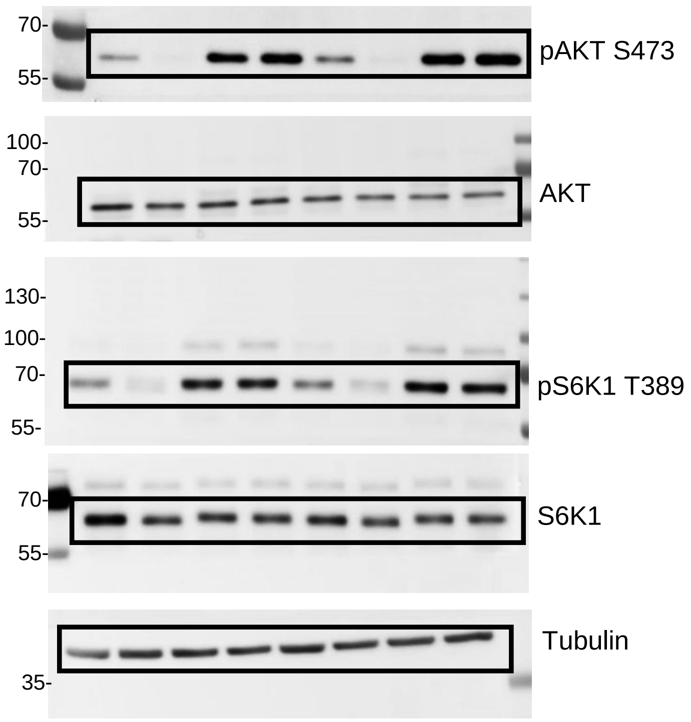

Fig. S3A

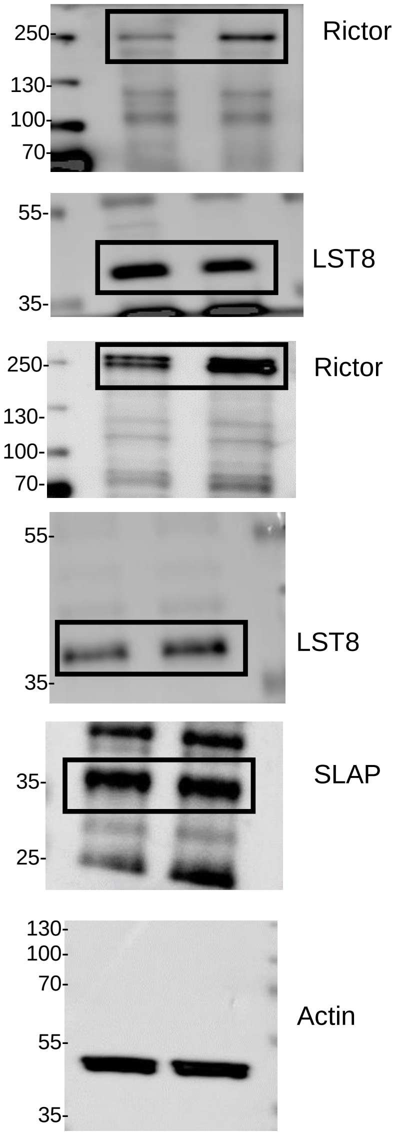

Fig. S3B

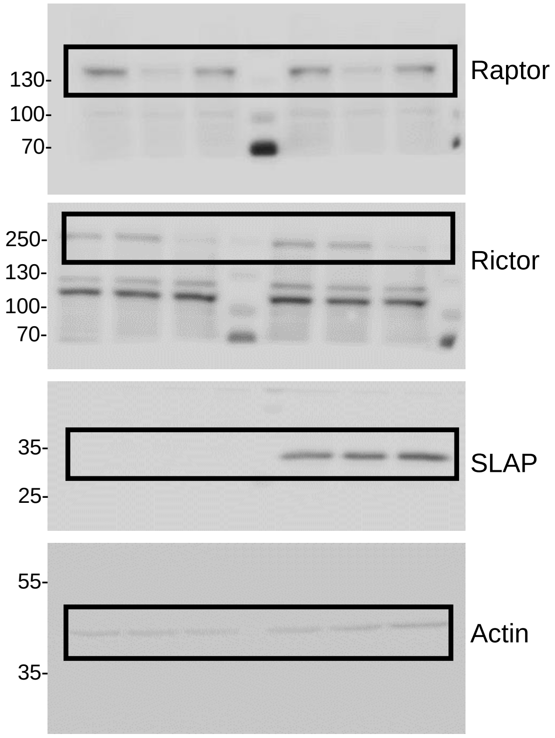

Fig. S4A

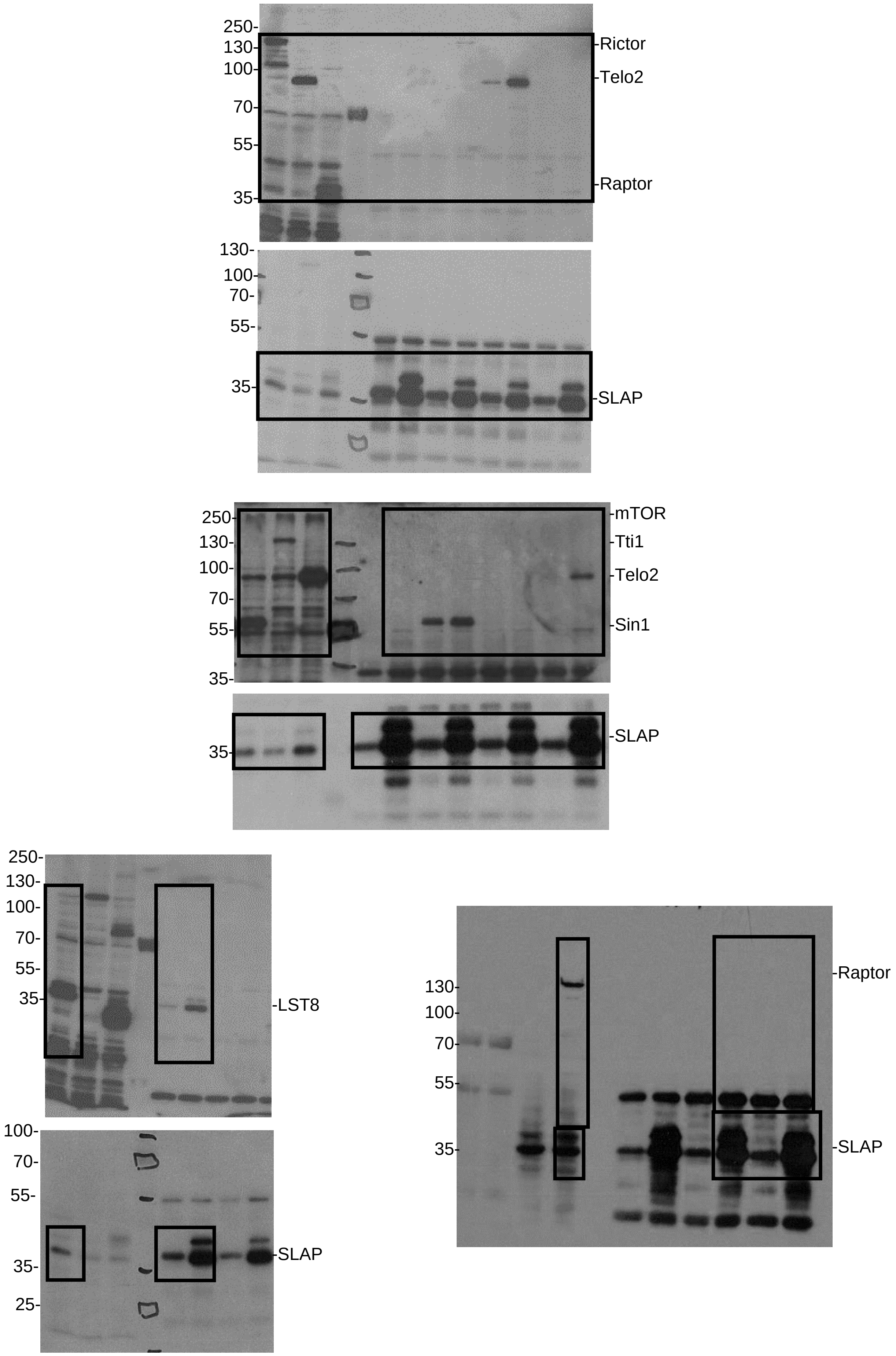
250-
130-
100-
70-
55-
35-
-Rictor
-Telo2
-Raptor
130-
100-
70-
55-
35-
-SLAP
250-
130-
100-
70-
55-
35-
-mTOR
-Tti1
-Telo2
-Sin1
35-
-SLAP
250-
130-
100-
70-
55-
35-
-LST8
100-
70-
55-
35-
25-
-SLAP
130-
100-
70-
55-
35-
-Raptor
-SLAP

Fig. S4B

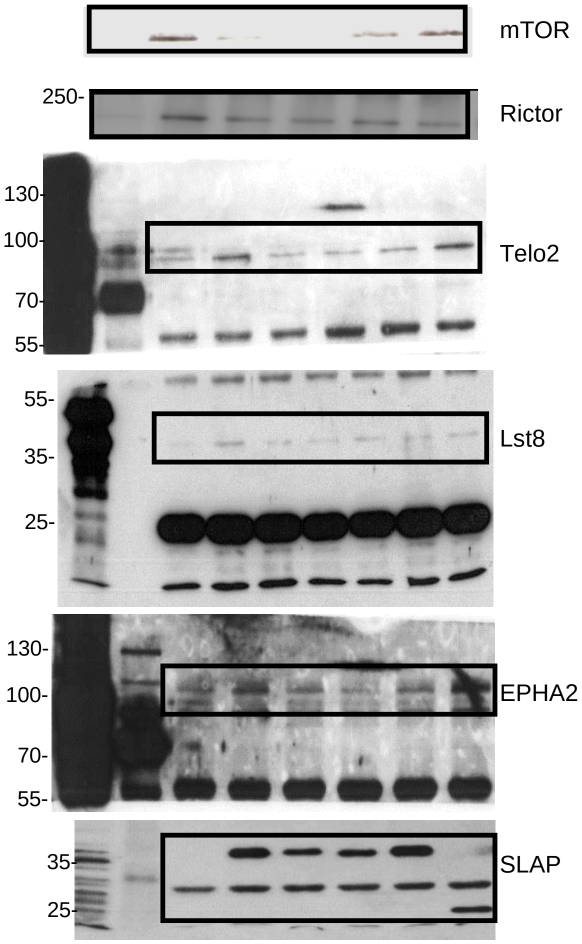

Fig. S4C

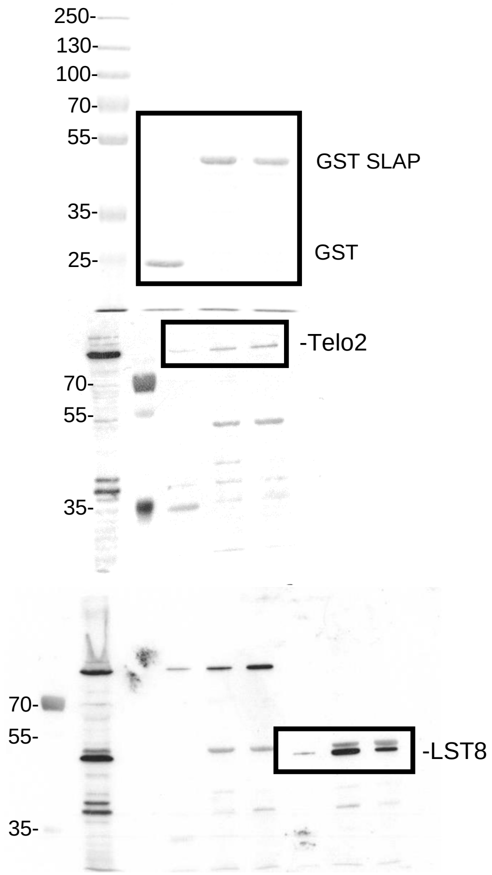

Fig. S4D

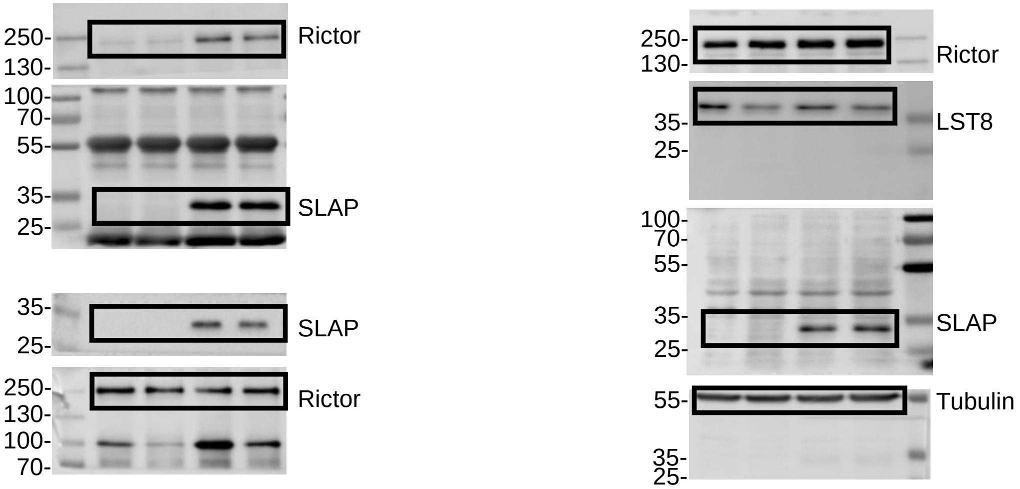

Fig. S5A

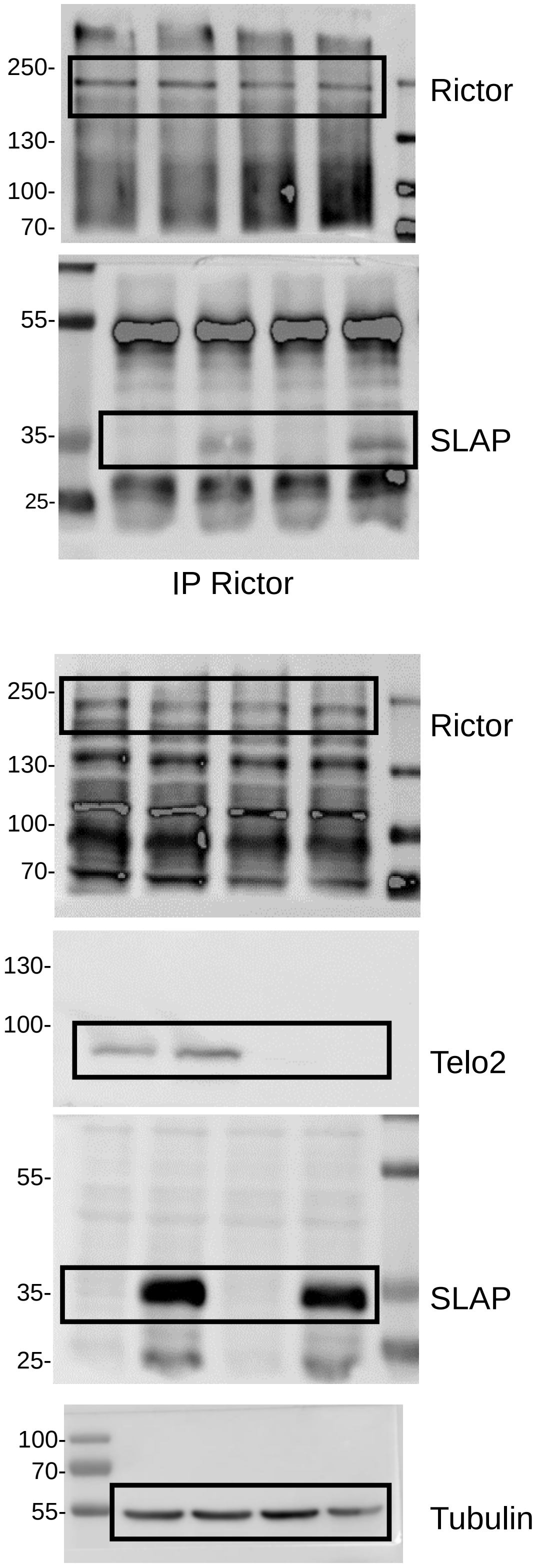

Fig. S5B

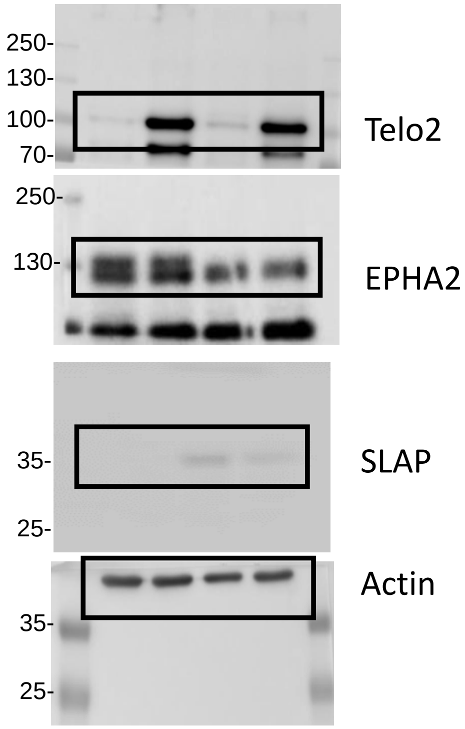

Fig. S6A

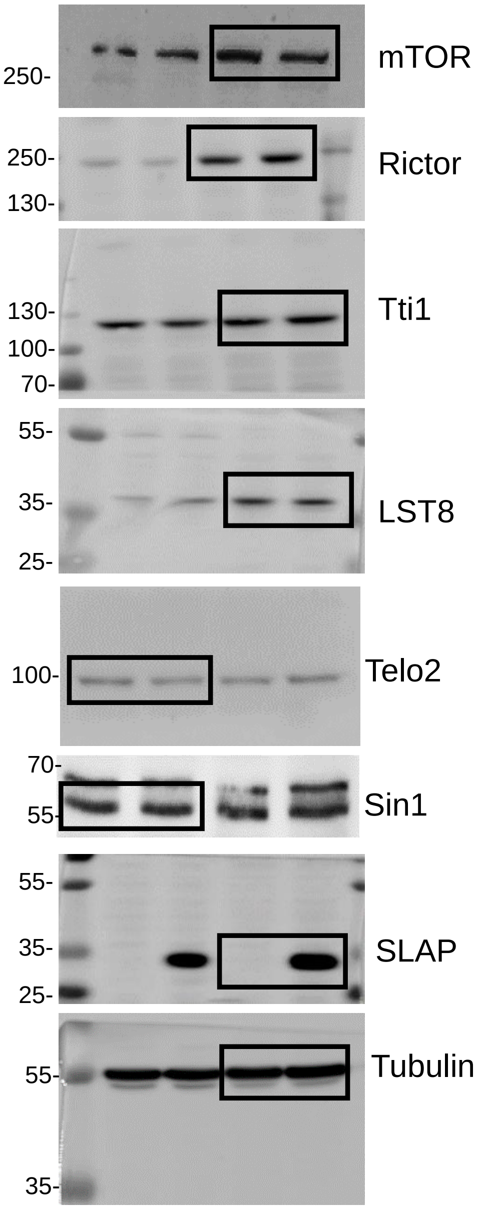

Fig. S6B

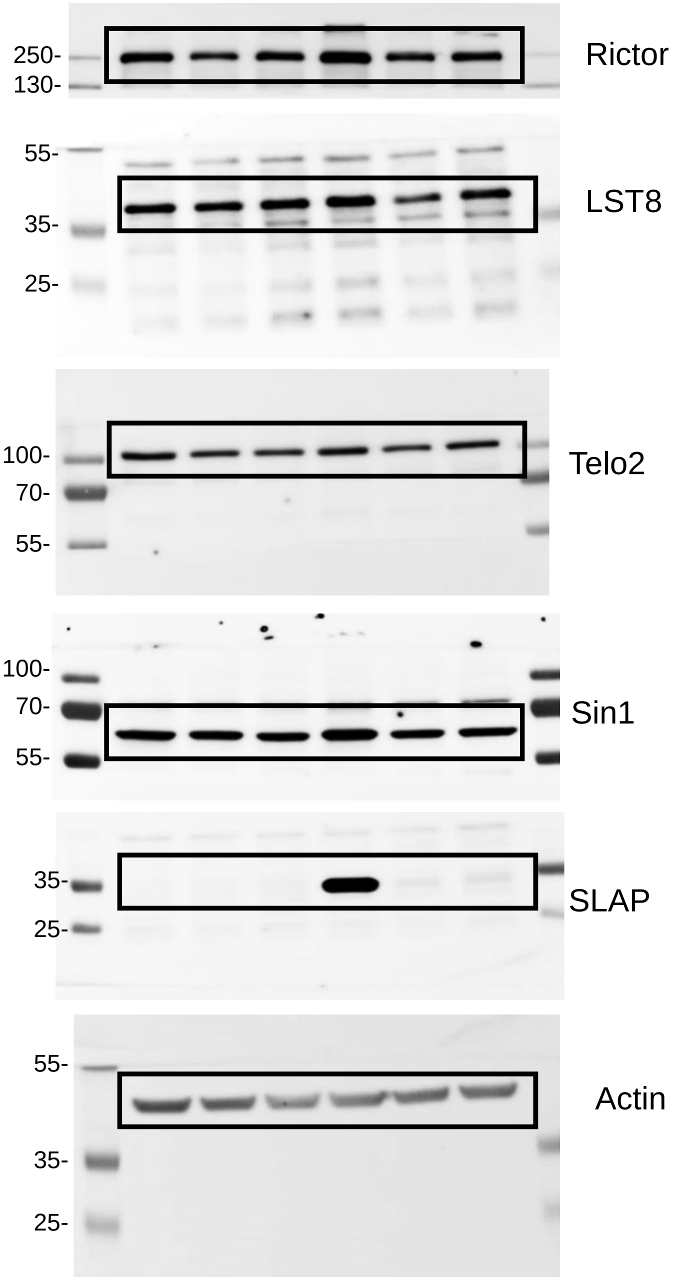

Fig. S7

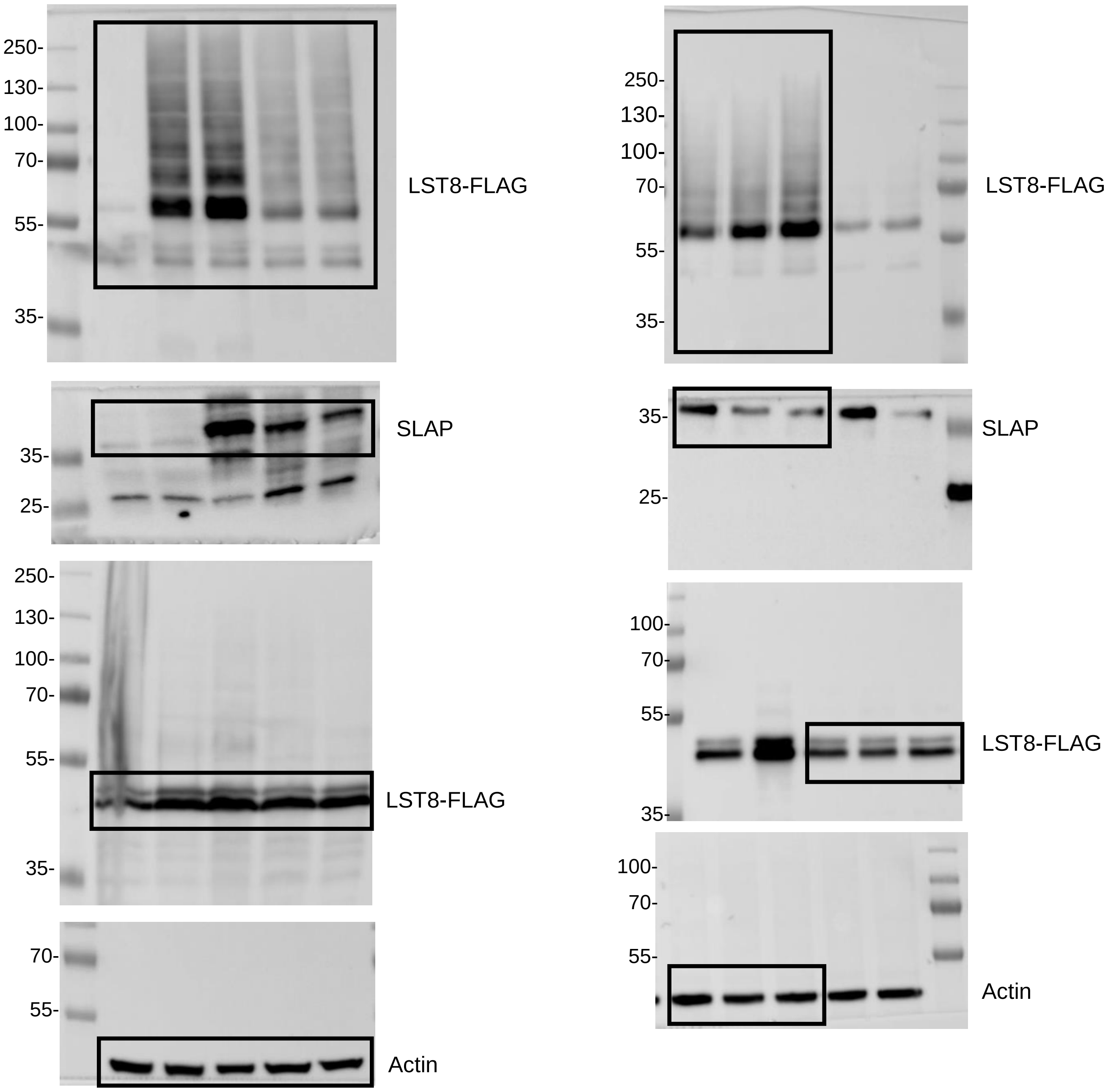

250-
130-
100-
70-
55-
35-
LST8-FLAG
35-
25-
SLAP
250-
130-
100-
70-
55-
35-
LST8-FLAG
70-
55-
Actin
250-
130-
100-
70-
55-
35-
LST8-FLAG
35-
25-
SLAP
100-
70-
55-
35-
LST8-FLAG
100-
70-
55-
Actin

Fig. S8

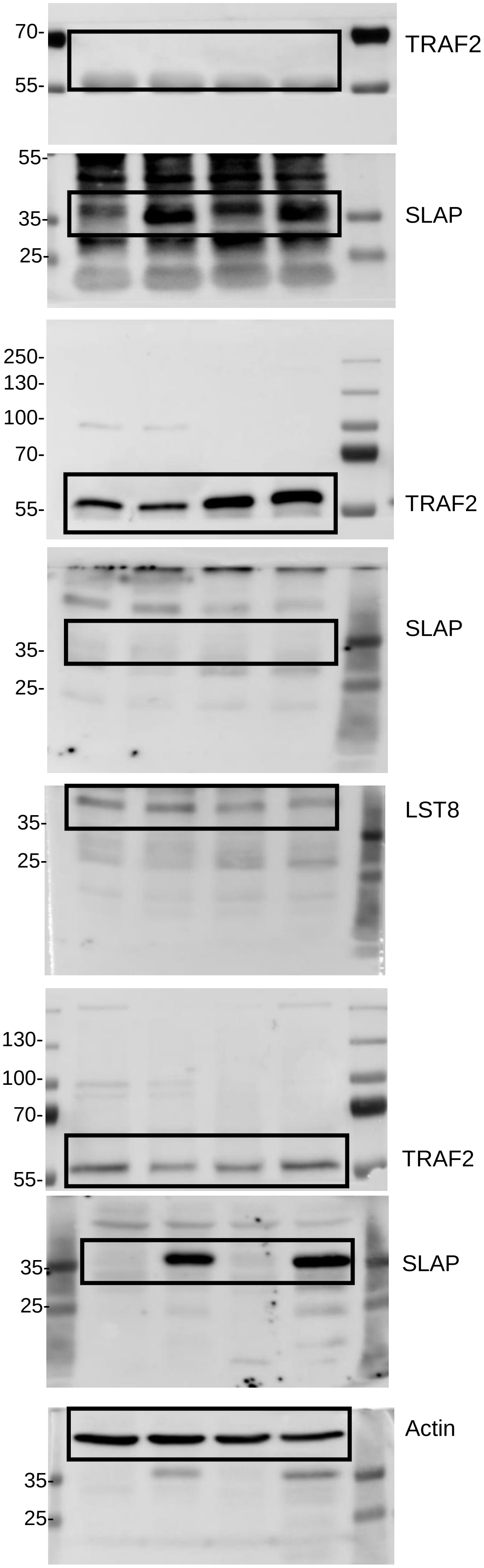
70-
55-
TRAF2
55-
35-
25-
SLAP
250-
130-
100-
70-
55-
TRAF2
35-
25-
SLAP
35-
25-
LST8
130-
100-
70-
55-
TRAF2
35-
25-
SLAP
35-
25-
Actin

Fig. S10A

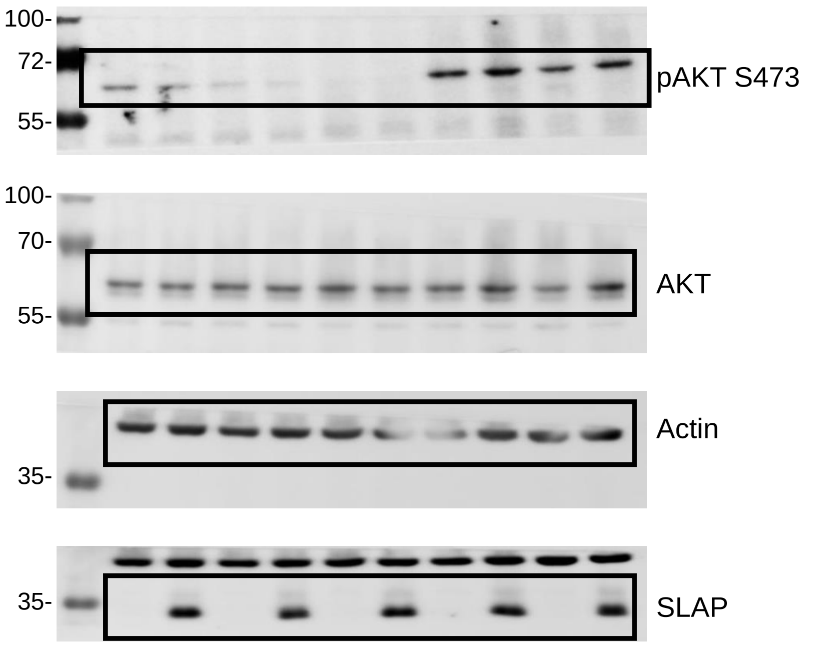

Fig. S10C

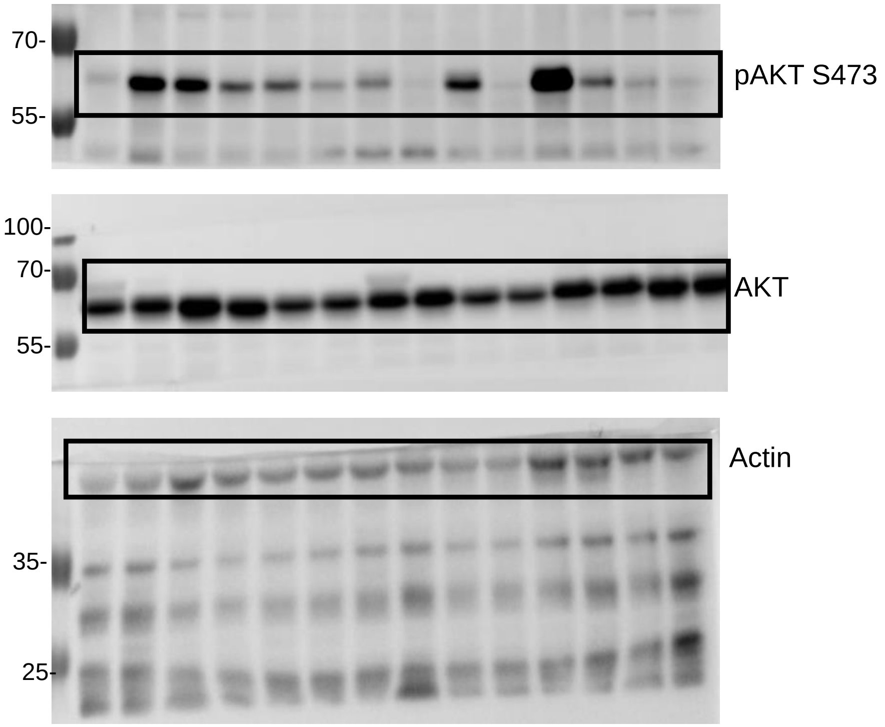

Supplement: Supplementary file 2 — Original blots [file 41418_2025_1633_MOESM2_ESM.pdf]
